# HLA-dependent heterogeneity and macrophage immunoproteasome activation during lung COVID-19 disease

**DOI:** 10.1186/s12967-021-02965-5

**Published:** 2021-07-05

**Authors:** Christophe Desterke, Ali G. Turhan, Annelise Bennaceur-Griscelli, Frank Griscelli

**Affiliations:** 1 INSERM UA9- University Paris-Saclay, 94800 Villejuif, France; 2 ESTeam Paris Sud, INGESTEM National IPSC Infrastructure, University Paris-Saclay, 94800 Villejuif, France; 3 Division of Hematology, Kremlin-Bicetre Hospital, 94270 Kremlin Bicetre, France; 4 University Paris Saclay, Faculty of Medicine, 94275 Le Kremlin Bicêtre, France; 5 University of Paris, Faculty Sorbonne Paris Cité, Faculté Des Sciences Pharmaceutiques Et Biologiques, Paris, France; 6 Department of Biopathology, Gustave-Roussy Cancer Institute, 94800 Villejuif, France; 7 INSERM UA9, Institut André Lwoff, Hôpital Paul Brousse, Bâtiment A CNRS, 7 rue Guy Moquet, 94802 Villejuif, France

**Keywords:** COVID-19, Macrophage, Immunoproteasome, HLA, Peptidome, Antigen presentation, Transcriptome, Integrative analysis, MHC class-I

## Abstract

**Background:**

The worldwide pandemic caused by the SARS-CoV-2 virus is characterized by significant and unpredictable heterogeneity in symptoms that remains poorly understood.

**Methods:**

Transcriptome and single cell transcriptome of COVID19 lung were integrated with deeplearning analysis of MHC class I immunopeptidome against SARS-COV2 proteome.

**Results:**

An analysis of the transcriptomes of lung samples from COVID-19 patients revealed that activation of MHC class I antigen presentation in these tissues was correlated with the amount of SARS-CoV-2 RNA present. Similarly, a positive relationship was detected in these samples between the level of SARS-CoV-2 and the expression of a genomic cluster located in the 6p21.32 region (40 kb long, inside the MHC-II cluster) that encodes constituents of the immunoproteasome. An analysis of single-cell transcriptomes of bronchoalveolar cells highlighted the activation of the immunoproteasome in CD68 + M1 macrophages of COVID-19 patients in addition to a PSMB8-based trajectory in these cells that featured an activation of defense response during mild cases of the disease, and an impairment of alveolar clearance mechanisms during severe COVID-19. By examining the binding affinity of the SARS-CoV-2 immunopeptidome with the most common HLA-A, -B, and -C alleles worldwide, we found higher numbers of stronger presenters in type A alleles and in Asian populations, which could shed light on why this disease is now less widespread in this part of the world.

**Conclusions:**

HLA-dependent heterogeneity in macrophage immunoproteasome activation during lung COVID-19 disease could have implications for efforts to predict the response to HLA-dependent SARS-CoV-2 vaccines in the global population.

**Supplementary Information:**

The online version contains supplementary material available at 10.1186/s12967-021-02965-5.

## Background

The pandemic caused by the SARS-CoV-2 coronavirus has become the chief public health challenge for many countries around the world. Respiratory complications have been well documented in patients with this disease (COVID-19). This coronavirus harbors a viral spike (S) protein which, during infection, binds with the human protein receptor ACE2 [1]; the virus’ entry into the cell and consequent infectivity is also facilitated by the host receptor neuropilin-1 (Cantuti-Castelvetri et al., 2020). The mean incubation period of the disease is about 3 to 9 days [2] and about 18% of cases remain asymptomatic [3]. The severity of the disease can increase with age, with the risk of mortality increasing in patients over 60 years old [4]. One of the potential consequences of SARS-CoV-2 infection is an uncontrolled immune response in the lungs, which can require treatment in an intensive care unit. This immune response, associated with a cytokine storm, is heterogenetic and variable among individuals, and is still not well understood [5]. The innate immune response is the first line of defense against invading microorganisms, and macrophages in particular play an important role in the response to respiratory tract infections. In the case of SARS-CoV-2, the host’s innate immune response uses a wide variety of pattern recognition receptors to recognize different types of highly conserved viral residues [6]. In vitro, human monocytes/macrophages were found to be induced by the coronavirus spike protein via activation of the NF-kappaB pathway [7]. In vivo, a dramatic increase in IL-6-producing CD14+ /CD16+ monocytes was observed in the peripheral blood of COVID-19 patients in the intensive care unit. Death from adult respiratory distress syndrome as a result of COVID-19 has been linked to a prolonged increase in IL-6 and IL-1-like cytokines that results in hyper-inflammation, known generally as cytokine storm or, more specifically, macrophage activation syndrome [8]. Extreme geographical variations have been observed in the prevalence of COVID-19 infection, with higher rates of infection in Europe and the Americas and lower rates in Asia, Africa, the Eastern Mediterranean, and the Western Pacific. Furthermore, emerging observations that a significant percentage of individuals are asymptomatic suggests not only that SARS-CoV-2 may have a longer incubation period and higher transmission rate than other coronaviruses, but also hints at potential differences in hosts’ immune responses to this virus.

In particular, an improved understanding of human T cell–mediated immunity in COVID-19 is important for optimizing therapeutic and vaccine strategies. In general, CD8+ or CD4+ T lymphocytes recognize different human leucocyte antigen (HLA)-peptide complexes via a mechanism called T-cell restriction. Restriction of CD8+ cytotoxic T lymphocytes is shaped by polymorphisms in exons 2 and 3 of HLA class I molecules, while CD4+ helper T cells are restricted via polymorphisms in exon 2 of HLA class II [9]. Macrophages, as part of the innate immunity system, have a key role at airway levelin the anti-viral response. This specific cell population participates in the antigen presentation and could be the target of cytotoxic T cells during infection [10]. CD8 cytotoxic response is activated through HLA class I antigen presentation mediated via the proteasome, which could be activated in the immunoproteasome after interferon-γ stimulation [11]. During the activation of this 20S proteasome, the catalytically active subunits β1c (PBSM6), β2c (PBSM7) and β5c (PBSM5) are replaced by MECL-1 (β2i) alias PBSM10, LMP2 (β1i) alias PBSM9, and LMP7 (β5i) alias PBSM8 initiating the proteolytical activation of subunits of the immunoproteasome [12], a special type of proteasome mainly expressed in hematopoietic cells. Immunoproteasome inhibition reduces the secretion of several proinflammatory cytokines such as IL-23 and specific inhibitors of LMP2/LMP7 selectively kills human CD14 + monocytes [13]. Also, immunoproteasome inhibition could alter alveolar macrophage polarization. Classical activation (M1 polarization) at transcriptional level of primary alveolar macrophages by LPS/IFNγ induced three immunoproteasome subunits: low molecular mass protein 2 (LMP2), LMP7 and multi-catalytic endopeptidase complex-like 1, which were accompanied by increased immunoproteasome activity in M1 cells. Alveolar macrophages from LMP7 knockout mice disclosed a distorted M2 profile upon IL-4 stimulation, characterized by increased M2 marker gene expression and CCL17 cytokine release [14].

In this work, we observed that MHC class I antigen presentation was strongly activated in the lungs of COVID-19 patients, and this activation reflected the prevalence of SARS-CoV-2 RNA. In the same samples, we detected a positive relationship between viral load and activation of the genomic cluster associated with the immunoproteasome. Further investigation of single-cell heterogeneity in bronchoalveolar cells revealed a cell trajectory in M1 macrophages during mild cases of COVID-19 that is shaped by expression of the PSMB8 subunit of the immunoproteasome. The single-cell transcriptomes of equivalent cells from severe COVID cases instead demonstrated an impairment of alveolar clearance mechanisms (e.g., lipidic transport and catabolism, MARCO scavenger receptor). Predictions of binding affinity between SARS-CoV-2 and some of the most prevalent MHC class I presenters worldwide identified a high diversity of strongly presenting alleles in Asian populations.

## Methods

### Bioinformatics analyses

Bioinformatics analyses were performed in the Linux-based operating system Ubuntu 20.04 LTS with the R software environment version 4.0.2. To assemble a dataset of immunoproteasome-related genes, genes were identified from the review of Ferrington and Gregorson (2012) via text mining with the Pubtator central algorithm [15]. Functional enrichment of targeted genesets was analyzed using the online Toppgene application [16]. The transparent bioinformatics code for all analyses performed in R software is provided at the address: https://github.com/cdesterke/covid19if.

### Transcriptome analyses of COVID-19 lung samples

We obtained transcriptomes from COVID-19 lung biopsies from dataset GSE150316 in the Gene Expression Omnibus database (https://www.medrxiv.org/content/10.1101/2020.07.30.20165241v1) [17]. A comparison of the transcriptomes between lung samples presenting high and low levels of SARS-CoV-2 RNA was performed with Gene Set Enrichment Analysis (GSEA) software version 4.0.3 [18] using version 7.2 of the MSigDB database [19]. A network of genes implicated in the functional enrichment in antigen presentation was created with Cytoscape standalone software version 3.6.0 [20]. Principal component analyses were performed with the R-package FactoMineR [21]. Correlated expression plots were created using the R-package corrgram. The genomic relationships among activated immunoproteasome components were visualized by loading version HG38 of the human genome in the Integrative Genomics Viewer version 2.3.40 [22]. Univariate clinical parameter analysis during stratification of the cohort between groups of patients presenting low and high levels of SARS-COV2 in lung were performed with Publish R-package.

### Single-cell analysis of lung samples from healthy donors and patients with mild and severe COVID-19

In order to validate, at the single-cell level, the disruption observed in immune molecules in the transcriptome analysis of lung cells from COVID-19 patients, single-cell transcriptome (10 × Genomics) data from bronchoalveolar lavage fluid samples were downloaded in H5 format from the dataset GSE145926 [23]. From this dataset, we created a merged matrix by aggregating a total of 90696 transcriptomes. This included six healthy donor samples—GSM4475048, GSM4475049, GSM4475050, GSM4475051, GSM4475052, GSM4475053—comprising 39900 transcriptomes; three samples from patients with mild COVID-19—GSM4339769, GSM4339770, GSM4339772—comprising 9710 transcriptomes; and three samples from patients with severe COVID-19—GSM4339771, GSM4339773, GSM4339774—comprising 41086 transcriptomes. Clinical stratification between mild and severe COVID-19 was performed in the original study. Moderate cases have fever, respiratory symptoms and pneumonia evidenced by computed tomography (CT) imaging. Patients with severe infection were diagnosed on the basis of one of the following criteria: (1) respiratory distress with respiratory rate ≥ 30 times min − 1; (2) fingertip oxygen saturation ≤ 93% at resting state; (3) ratio of partial pressure of arterial oxygen to fraction of inspired oxygen (PaO2/FiO2) ≤ 300 mm Hg (1 mm Hg = 0.133 kPa); and (4) obvious progression of lesions in 24–48 h shown by pulmonary imaging > 50% [23].

After canonical correlation and scaling, a total of 23,742 features were analyzed, with 38738 anchors identified between samples. After variable feature selection with the VST algorithm, dimensionality reduction was carried out by principal component analysis on 2000 variable features (30 components) and UMAP dimensionality reduction was performed on the 20 best components of the PCA. Single-cell analyses of the CD14+/CD68+ subset of cells were performed in Seurat, and PSMB8-dependent trajectories were constructed for the CD14+/CD68+ subset with the R-package monocle2.

### MHC class I immunopeptidome of SARS-CoV-2 virus

Protein sequences corresponding to the totality of the proteome of the Wuhan-Hu-1 isolate of SARS-CoV-2 were downloaded from the NCBI nucleotide database at the following address: https://www.ncbi.nlm.nih.gov/nuccore/1798174254. Data on the prevalence of HLA alleles in 20 ethnicities from the American bone marrow registry were collected from the HLA Allele Frequency net database; USA NMDP bone marrow registry database available at the address: http://www.allelefrequencies.net/ [24]. The distributions of HLA alleles among world regions were predicted by unsupervised analysis and validated by Random Forest estimation of error of bagging [25]. Immunopeptidome binding affinity was predicted for each MHC class I binding pocket with the algorithm NetMHCpan class I version 4.1 [26]. Predicted relationships between peptides and HLA alleles were divided into three subgroups: weak, with binding affinities estimated between 500 and 5000 nM; regular, with binding affinities estimated between 50 and 500 nM; and strong, with binding affinities estimated under 50 nM. A heatmap was created to represent the relative binding activities of MHC class I alleles using the R-package pheatmap, with Euclidean distances and the Ward.D2 method. Cleveland plots of HLA prevalence in different ethnic groups and barplots of peptide binding were created with the R-library ggpubr.

## Results

### Activation of MHC class I antigen presentation in the lungs of COVID-19 patients is associated with the amount of SARS-CoV-2 RNA in the tissue

**Fig. 1:**
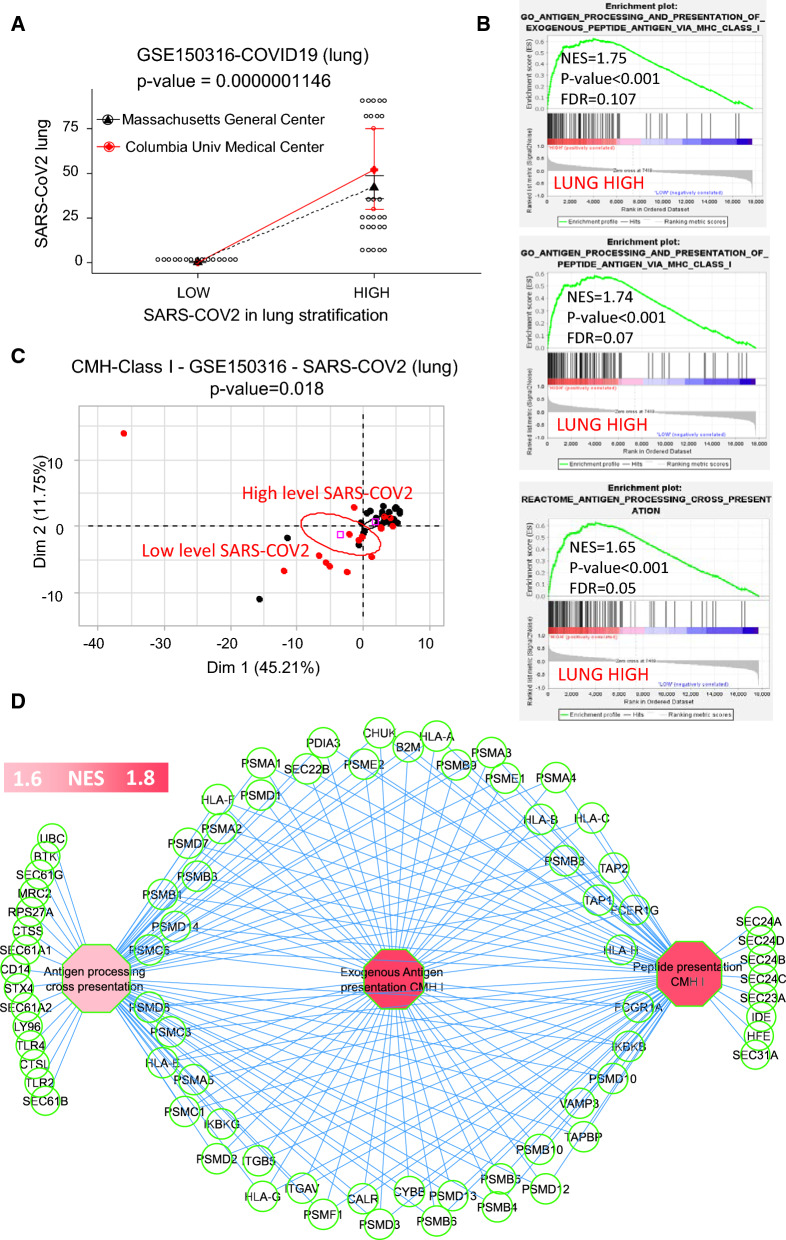
Activation of MHC class I antigen presentation in lung tissue of COVID-19 patients is associated with amount of SARS-CoV-2 present in the tissue. **A** Dot plot of SARS-COV2 lung quantification stratified on patients groups and medical centers (two sided ttest p-value for group stratification). **B** Geneset enrichment analysis demonstrating relative expression of MHC class I antigen processing and presentation machinery in lung samples with high levels of SARS-CoV-2 *versus* lung samples with low levels of SARS-CoV-2 (NES: normalized enrichment score, FDR: False Discovery Rate). **C** Unsupervised principal component analysis of expression profiles of genes associated with MHC class I antigen presentation, with clustering patterns reflecting the levels of SARS-CoV-2 detected in the lung (Pearson p-value was estimated for group discrimination along the first principal axis). **D** Functional network of genes implicated in the SARS-CoV-2–associated activation of MHC class I antigen presentation in lung tissue of COVID-19 patients. All analyses were performed using transcriptome dataset GSE150316

A certain proportion of COVID-19 patients admitted to hospitals develop acute respiratory distress syndrome [27]. We thus decided to investigate the lung transcriptomes of patients with COVID-19 using gene-set enrichment analysis (GSEA) to identify differences between two groups of patients who presented different levels of SARS-CoV-2 virus in their tissues, as detected by in situ hybridization (ISH) of viral RNA. A cohort of 14 patients was screened using 45 different microarray experiments in lung samples and their SARS-COV2 levels were also assessed. So, a two-group stratification of GSE150316 transcriptome cohort was defined according to their level of SARS-COV2 in lung (threshold 5 percent). Patients processed in this cohort come from two different medical centers: a majority of them from the Massachusetts General Hospital and a minority from the Colombia university Medical center (Fig. 1A). Clinical analysis of this cohort stratification revealed a significant association of SARS-COV2 virus quantification between the two groups of patients with a positive association (p-value = 6.67E−4, Additional file 1: Table S1). Analysis performed on symptoms at admission showed that patients that harbored high levels of virus in the lung were older with less coughing and myalgia but had increased symptoms of lethargy, weakness and hypotension (Additional file 1: Table S1). The preexisting disease association analysis, showed a negative relation between hypertension and high levels of SARS-COV2 but suggested a positive relation between gastrointestinal reflex diseases and high level of SARS-COV2 (Additional file 1: Table S2). Time from admission to death was shorter for patients with high level of SARS-COV2 (3 days versus 14 days, p = 0.0029) showing that this stratification in two groups was clinically justified. Consequently these patients were hospitalized for a shorter period of time than patients with a low viral load (8 days versus 17 days, p = 0.0082). Therapy intervention analysis showed a tendency for a negative relation between administrations of atorvastatin and hydroxychloroquine suggesting that these treatments tend to be more administrated in group of patients with low level of virus (Additional file 1: Table S3). An analysis of gene set enrichment was conducted using MSigDB version 7.2 with the Reactome and Gene Ontology subset; this revealed a significant increase in MHC class I antigen processing and presentation in the cells of patients with higher viral loads (NES: normalized enrichment score, FDR: False Discovery Rate, Fig. 1B). A principal component analysis was carried out on the expression profiles of genes associated with MHC class I antigen processing and presentation, and the resulting clustering pattern significantly discriminated between lung samples with a low amount of virus and those with a high amount of virus (p-value = 0.018, Fig. 1C). Using these expression profiles, we were also able to build a highly connected gene (174 relations) network for MHC class I antigen processing and presentation (Fig. 1D, Additional file 1: Table S4). These results suggest that in the lungs of COVID-19 patients, the intensity of antigen processing and presentation by class I molecules is proportional to the level of detected virus.

### Activation of genomic cluster associated with the immunoproteasome in the lungs of COVID-19 patients correlates with the amount of SARS-CoV-2 RNA in the tissue

**Fig. 2:**
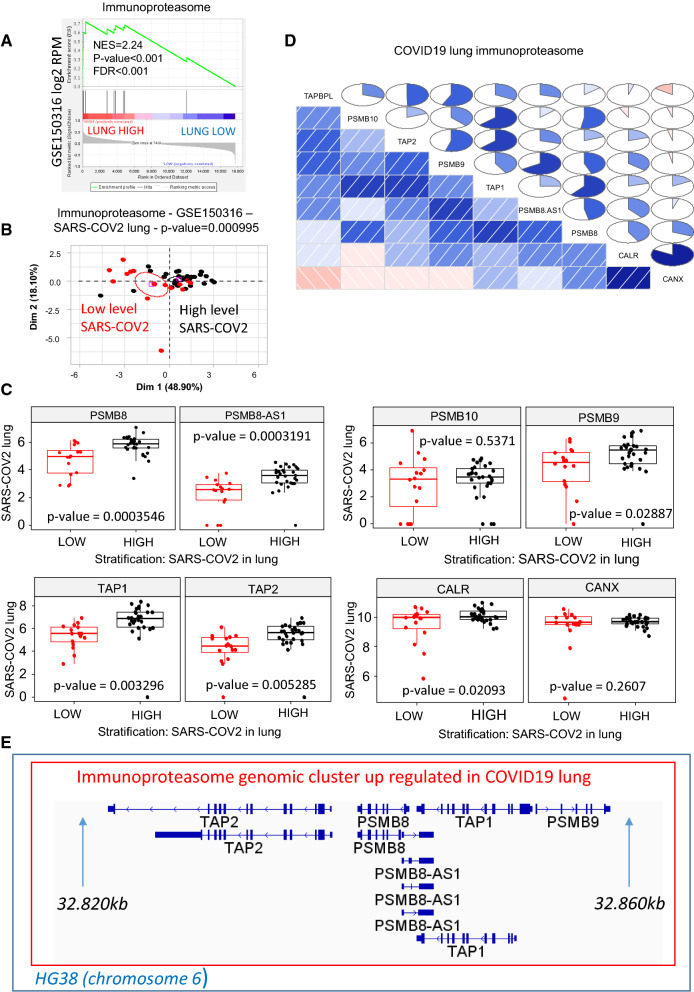
Genomic activation of the immunoproteasome in lung tissue of COVID-19 patients correlates with the amount of SARS-CoV-2 detected. **A** Geneset enrichment analysis demonstrating the relative expression of the genomic cluster of the immunoproteasome in lung samples with high levels of SARS-CoV-2 versus lung samples with low levels of SARS-CoV-2 (NES: normalized enrichment score; FDR: False Discovery Rate). **B** Unsupervised principal component analysis of expression profiles of immunoproteasome-associated genes, with clustering patterns reflecting the level of SARS-CoV-2 detected in the lung (Pearson p-value was estimated for group discrimination along the first principal axis). **C** Boxplots of immunoproteasome-related gene expression as a function of the level of SARS-CoV-2 detected in the lung (two sided ttest p-values between low and high groups). **D** Correlation plot illustrating the relationship between lung expression of different immunoproteasome-related genes in patients with COVID-19 (blue: positive correlation, red: negative correlation). **E** Location of immunoproteasome genes that demonstrated SARS-CoV-2–associated activation on chromosome 6 (HG38). All analyses were performed using transcriptome dataset GSE150316

During MHC antigen presentation, catalytic subunits of the immunoproteasome are tightly regulated in different cell types, leading to the production of distinct repertoires of presented peptides [28]. To assess potential changes in the expression of such genes in COVID-19 patients, we first assembled a geneset of immunoproteasome-associated genes. Using the PubTator Central text-mining algorithm [15], which explores the full text of articles in Pubmed Central, we analyzed all gene citations in Ferrington and Gregerson’s [28] review of immunoproteasome literature (PMC4405001). The resulting immunoproteasome-related gene set was then used to conduct a GSEA analysis between the two groups of COVID-19 patients (GSE150316) with low and high levels of virus in the lung. With this, we detected an increase in immunoproteasome activation that followed the level of virus detected in the lung (NES =  + 2.24, Fig. 2A). Unsupervised principal component analysis performed with immunoproteasome related genes confirmed stratification of patient groups with low and high levels of SARS-COV2 in the lungs (p-value = 0.00099, Fig. 2B). To further investigate immunoproteasome implication, we performed differential expression analysis of its main components between groups of patients with low and high levels of SARS-COV2 virus in the lung. Specifically, the amount of virus in lung tissue was found to be associated with an increase in the expression of the following genes: PSMB8 (p-value = 0.00035, Fig. 2C), PSMB8-AS1 (antisense of PSMB8, p-value = 0.00032, Fig. 2C), PSMB9 (p-value = 0.029, Fig. 2C), TAP1 (p-value = 0.0033, Fig. 2C), TAP2 (p-value = 0.0053, Fig. 2C) and CALR (p-value = 0.021, Fig. 2C). When we examined the expression of immunoproteasome-related genes in the transcriptome dataset, we observed a positive correlation between the expression of PSMB8 and TAP1, CALR and CANX, TAP1 and TAP2, and TAP1 and PSMB10 (Fig. 2D). Co-regulated genes of the immunoproteasome were found to map together to the 6p21.32 genomic cluster of the human genome (HG38), specifically, inside the HLA class II region, from 32.820 to 32.860 kb (Fig. 2E). These results suggest that in the lungs of COVID-19 patients, immunoproteasome components located in a 40-kb span of the 6p21.32 genomic region are activated in a manner that may be dependent on the viral load of SARS-CoV-2.

### Single-cell transcriptomes of bronchoalveolar cells reveal immunoproteasome activation in M1 macrophage cells during mild cases of COVID-19

**Fig. 3:**
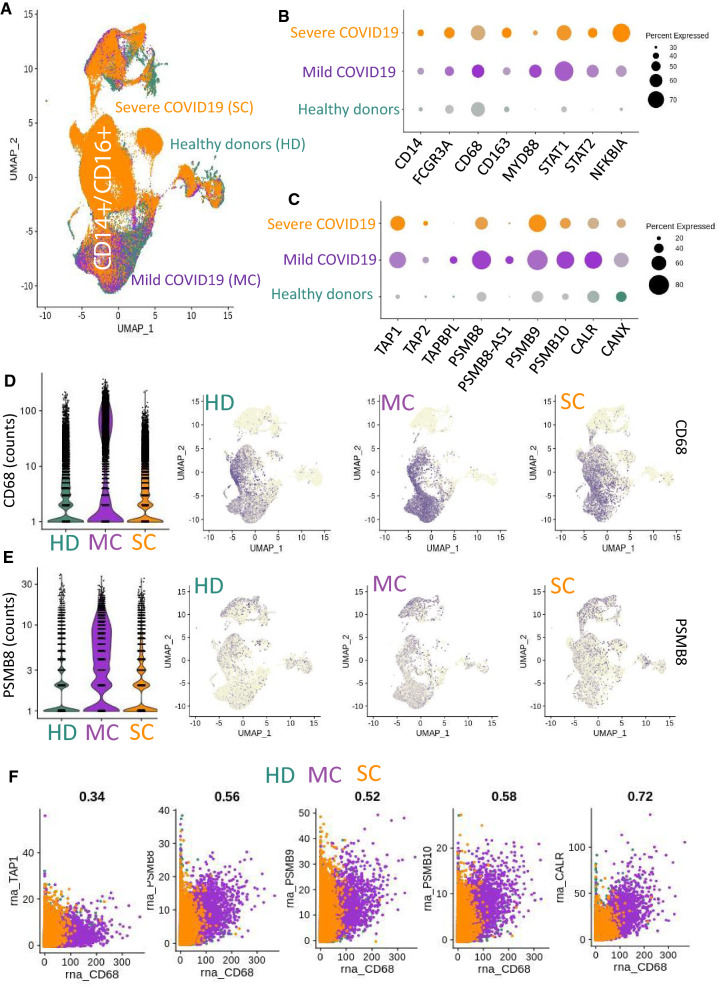
Analysis of single-cell heterogeneity in bronchoalveolar cells highlights activation of the immunoproteasome in M1 macrophages during mild COVID-19. **A** UMAP dimensionality reduction performed on gene expression in bronchoalveolar cells from healthy donors (HS, n = 6 subjects), patients with mild COVID-19 (MC, n = 3 patients), and patients with severe COVID-19 (SC, n = 3 patients). **B** Dotplot representing bronchoalveolar expression of macrophage and immune-signaling markers. **C** Dotplot representing bronchoalveolar expression of immunoproteasome-related genes. **D** Violinplot (left) and UMAP plot of single-cell bronchoalveolar expression (right) of the M1 macrophage marker CD68 among sample groups. **E** Violinplot (left) and UMAP plot of single-cell bronchoalveolar expression (right) of immunoproteasome subunit PSMB8 among sample groups. **F** Correlations between single-cell expressions of immunoproteasome-related genes *versus* that of M1 macrophage marker CD68 in bronchoalveolar cells

Next, patterns of immunoproteasome activation were investigated in a dataset of single-cell transcriptomes obtained from bronchoalveolar fluid lavages from six healthy donors, three patients with mild COVID-19, and three patients with severe COVID-19 [23]. These data were merged together for the purpose of UMAP dimensionality reduction (Fig. 3A) using the R-package Seurat [29]; after canonical correlation and filtration, the merged transcriptome analysis comprised 90,696 cells, with a representative proportion of each cell subgroup. A dotplot of the relative expression of macrophage-related markers appears in Fig. 3B. In these bronchoalveolar samples, we found that the M1 macrophage marker CD68, was expressed with a higher level in a cell population smaller in size for mild COVID-19 patients as compared to severe COVID-19 patients. As well as the macrophage-related genes MYD88 and STAT1, were upregulated in bronchoalveolar cells from patients with mild COVID-19, while cells from patients with severe COVID-19 were characterized by upregulation of the CD163 marker of M2 macrophages (Fig. 3B). Mild cases of COVID-19 were also characterized by increased expression of immunoproteasome components (Fig. 3C). Specifically, along with CD68 (Fig. 3D), the immunoproteasome-related genes PSMB8 (Fig. 3E), TAP1, PSMB9, PSMB10, and calreticulin (CALR) (Additional file 1: Figure S1) were all found to be expressed in the CD14+/CD16+ subpopulation of cells during mild COVID-19 (Fig. 3A). A correlation study of single-cell expression patterns in these bronchoalveolar cells revealed positive correlations between CD68 and TAP1 (coefficient r = 0.34), CD68 and PSMB8 (coefficient r = 0.56), CD68 and PSMB9 (coefficient r = 0.52), CD68 and PSMB10 (coefficient r = 0.58), and CD68 and CALR (coefficient r = 0.72) (Fig. 3F). When we instead investigated the relationships between these genes and the M2 macrophage marker CD163, which was overexpressed in bronchoalveolar cells from patients with severe COVID-19 (Fig. 3B and Additional file 1: Figure S2A), we found correlations that were still positive but notably weaker (between CD163 and PSMB8, coefficient r = 0.45; CD163 and PSMB9, coefficient r = 0.46; CD163 and PSMB10, coefficient r = 0.47; CD163 and CALR, coefficient r = 0.58) (Additional file 1: Figure S2B). These results suggest that immunoproteasome activation occurs in bronchoalveolar M1 macrophages during mild COVID-19 disease.

### Single-cell heterogeneity in PSMB8 expression shapes a cell trajectory in alveolar M1 macrophages during mild COVID-19

**Fig. 4:**
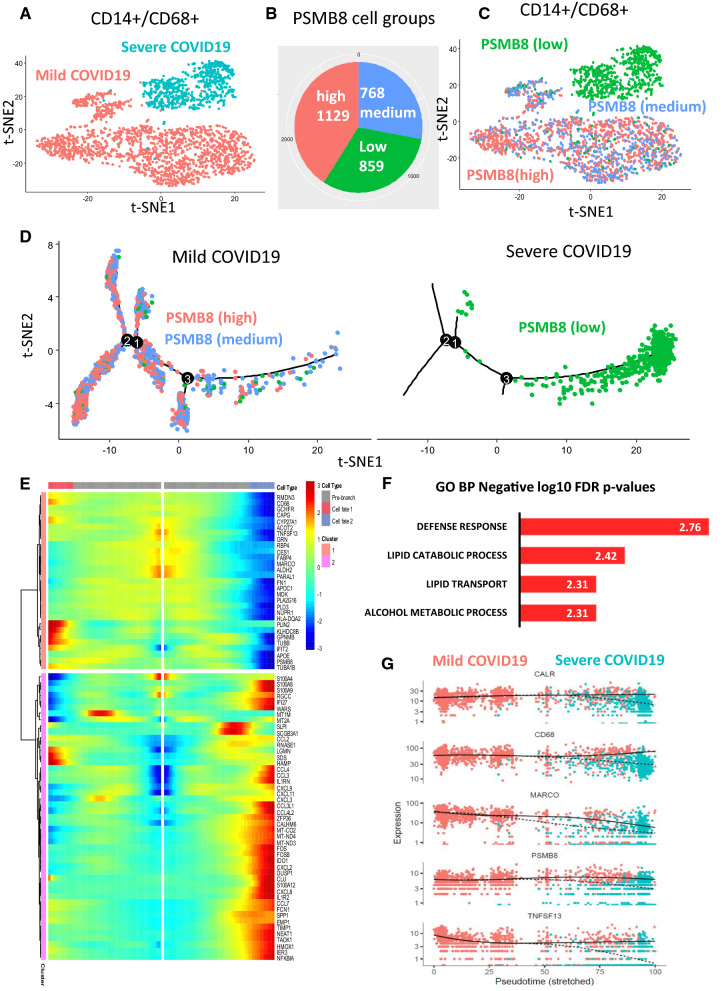
Single-cell heterogeneity in PSMB8 expression reveals cell trajectory in alveolar M1 macrophages during mild COVID-19. **A** t-SNE dimensionality reduction performed on transcriptomes of CD14+/CD68+ double-positive bronchoalveolar cells from COVID-19 patients. **B** Relative abundance of CD14+/CD68+ bronchoalveolar cells grouped according to level of PSMB8 expression during COVID-19 disease. **C** t-SNE dimensionality reduction performed on transcriptomes of CD14+/CD68+ bronchoalveolar cells from COVID-19 patients, with level of PSMB8 expression indicated by color; **D** Pseudotime transformation of cell trajectory based on PSMB8 expression in CD14+/CD68+ bronchoalveolar cells from COVID-19 patients. **E** Pseudotime trajectory expression heatmap of genes whose expression in CD14+/CD68+ cells from COVID-19 patients was linked to the PSMB8-based branch (identified with BEAM3 function in monocle R-package). **F** Assessment of gene ontology biological process (GO BP) functional enrichment in genes associated with the PSMB8 expression trajectory in CD14+/CD68+ bronchoalveolar cells from COVID-19 patients. **G** Expression dotplot of markers following the PSMB8 trajectory in CD14+/CD68+ bronchoalveolar cells from COVID-19 patients

Alveolar macrophages play a critical role in regulating immune responses [30] and maintaining homeostasis in the lung [31, 32]. Macrophages have been categorized as being “polarized” towards either the M1 (CD68+) proinflammatory phenotype, which mediates resistance to pathogens, or the M2 (CD163+) anti-inflammatory phenotype, which promotes tissue remodeling [33]. Using the same dataset of single-cell transcriptomes of bronchoalveolar cells in patients with mild and severe COVID-19 [23], we decided to examine the relationship between CD68+ macrophages and immunoproteasome components in more detail. With the R-package monocle [34], we constructed a cell trajectory of PSMB8 expression in the CD14+/CD68+ subpopulation of bronchoalveolar cells from COVID-19 patients (Fig. 4A–C). In the resulting pseudotime transformation of cell development, branch number 3 appears to differentiate between cells with low expression of PSMB8 (i.e. severe COVID-19) from those with medium and high levels of PSMB8 (mild COVID-19) (Fig. 4D). The pseudotime expression signature of this branch highlighted a major cluster of inflammation-associated genes that are activated in severe COVID-19 (bottom cluster, Fig. 4E) and a smaller cluster of molecules activated only in cells from mild cases of COVID-19 (top cluster, Fig. 4E). This latter group comprised both PSMB8 and the CD68 marker (Fig. 4E). When we analyzed functional enrichment in this gene cluster using the Toppgene internet application [16], we discovered that, in mild cases of COVID-19, CD68+  cells with high levels of PSMB8 appear to demonstrate enrichment in functions such as defense response (comprising PSMB8, CD68, GRN, APOE, FN1, MDK, NUPR1, IFIT2, MARCO, HLA-DQA2,CAPG, FABP4, and TUBB) and lipid homeostasis (lipid transport and lipid catabolism: APOC1, APOE, CES1, PLD3, FABP4, CES1, PLIN2, FABP4, RBP4, and CYP27A1) (Fig. 4F). Similarly, in the trajectory signature for alveolar macrophages from mild COVID-19 patients (Additional file 1: Table S5), CALR, MARCO and TNFSF13 followed the expression patterns of CD68 and PSMB8 (Fig. 4G). These results suggest that CD14+/CD68+ bronchoalveolar cell activation in mild COVID-19 is accompanied by enhanced expression of immunoproteasome-related components and functionalities implicated in the defense response and lipid homeostasis. Instead, all of these activities appeared to be impaired in the corresponding cell population in patients with severe COVID-19.

### High diversity in Asian populations for strong MHC class-I presenters of SARS-CoV-2

One of the main consequences of the activation of M1 macrophages is the presentation of viral peptides to the immune system and stimulation of the Th1 cytotoxic cell response. The efficiency of this mode of antigen presentation is still not well understood, but may be influenced by the genetic heterogeneity of presenters belonging to HLA class I. Of these, the genes HLA-A, -B, and -C are expressed in most human cell types and are the most polymorphic genes in the human genome, with over 12,000 distinct alleles documented worldwide. Other HLA class I molecules, like HLA-E, -F, and -G, are expressed only in specialized cell types [35]. We decided to examine the diversity of the most common HLA class I alleles (types A, B, and C) in order to predict which types might bind more effectively with SARS-CoV-2 peptides. First, we investigated the prevalence of the 69 most common HLA class I alleles (types A, B, and C only) worldwide [36]. This was accomplished through use of the bone marrow registry dataset from the USA, which documents the HLA status of 2,790,874 donors, representing 20 ethnicities with worldwide distribution (Additional file 1: Table S6) [24]. In total, 69 alleles were found to be prevalent in the 20 ethnic groups examined. An unsupervised principal component analysis based on the prevalence of these 69 alleles clustered the 20 ethnicities into three main groups, representing three major regions of the world: Africa, Asia, and Europe/Americas (Additional file 1: Figure S3A-B). These results suggest that these 69 alleles of HLA-A-B-C are representative of much of the world’s population diversity. Certain alleles demonstrated region-specific patterns of prevalence, such as HLA-C*08:01, with a prevalence of more than 0.15 in some Asian populations (Additional file 1: Figure S3C); HLA-A*25:01, with a prevalence of more than 0.20 in some Western populations (Additional file 1: Figure S3D); and HLA-B*53:01, with a prevalence of more than 0.10 in African populations (Additional file 1: Figure S3E). Other HLA alleles, instead, demonstrated more of a worldwide distribution (Additional file 1: Figure S3B). This pattern of clustering into three world regions based on allele prevalence was further validated by a Random Forest learning algorithm (100 trees, total error rate 0.23, Additional file 1: Figure S4A), which was successfully able to classify each allele/world region in the majority of cases (Additional file 1: Figure S4B–D). Furthermore, based on their respective scores for Accuracy and Gini Importance, ethnicities from each world region were found to contribute to the learning process (Additional file 1: Figure S4E), suggesting that these 69 HLA alleles were relatively evenly distributed worldwide.

**Fig. 5:**
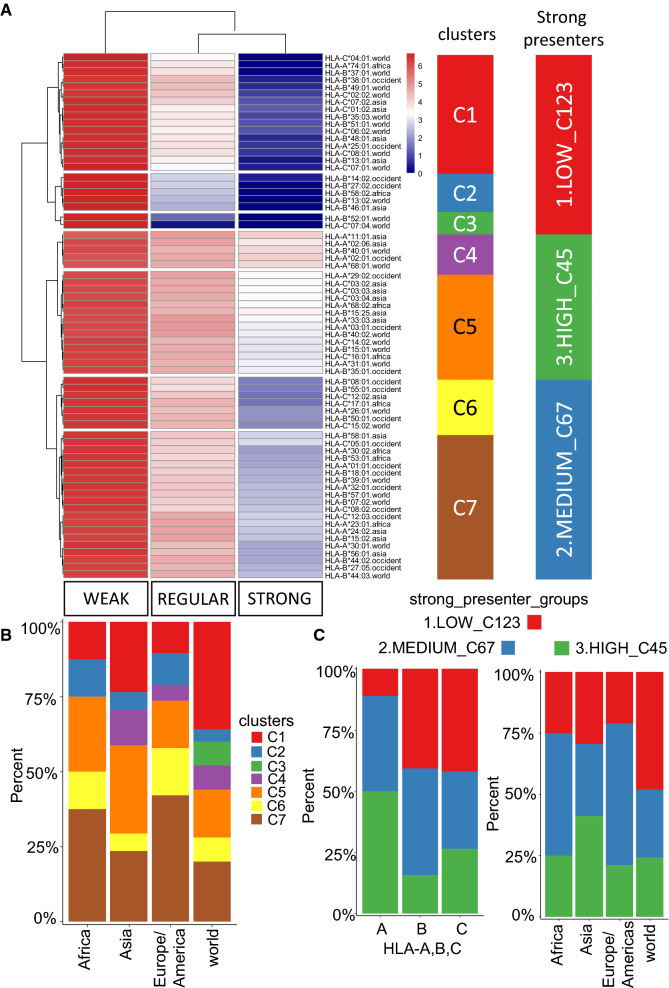
Asian populations contain a high diversity of MHC class I molecules that strongly bind the SARS-CoV-2 virus**. A** Unsupervised classification (Euclidean distances) of 69alleles of HLA-A, -B, or -C (all with prevalence higher than 0.01) based on their binding affinity for the SARS-CoV-2 immunopeptidome (three categories: strong 0–50 nM, regular 50–500 nM, weak 500–5000 nM). Color annotation depicts 7 allele clusters based on patterns of antigen presentation and 3 superclusters distinguished by proportions of strong presenters (low, medium, high). **B** Barplot of the prevalence of HLA clusters in populations from different world regions. **C** Barplot of supercluster representation among different types of HLA class-I alleles (**A**–**C**) and world regions

Our next step was to predict the respective binding patterns of these alleles to SARS-CoV-2 peptides using the algorithm NetMHCpan-4.1 [26], which is able to predict peptide binding to any MHC class I molecule of known sequence using artificial neural networks trained with information from 850,000 peptides (data on binding affinity and/or from mass spectrometry). For this analysis, we collected 38 protein sequences encoded by the entire genome of SARS-CoV-2 Wuhan-Hu-1 from the NCBI database (Additional file 1: Table S7) [37]. An analysis of these 38 protein sequences and the 69 tested alleles predicted 102,524 possible peptide binding events (Additional file 1: Table S8). As described by the literature associated with the NetMHCpan algorithm [26] and applied in the study of Barquera et al. [38], it is possible to distinguish three subgroups of MHC class I molecules based on their binding affinities: strong peptide binders, with a binding affinity between 0 and 50 nM; regular peptide binders, with a binding affinity between 50 and 500 nM; and weak peptide binders, with a binding affinity between 500 and 5000 nM. Peptide counts for the three binding subgroups and the 69 investigated HLA alleles are provided in Additional file 1: Table S9; based on their proportions, we were able to identify seven clusters of alleles (Fig. 5A). These seven clusters could be further grouped into three superclusters based on the proportion of strong binder peptides (Fig. 5A): cluster 1.LOW_C123 (red cluster), with low amounts of strong binder peptides; cluster 2.MEDIUM_C67 (blue cluster), with medium amounts of strong binder peptides; and cluster 3.HIGH_C45 (green cluster), with high amounts of strong binder peptides. When we examined the distribution of these clusters among world regions, we observed that clusters C6 and C7 (MEDIUM supercluster) were more prevalent in African and Western populations (Fig. 5B). In contrast, Asian populations harbored higher proportions of clusters C4 and C5, in the HIGH supercluster (Fig. 5B). This supercluster was largely composed of HLA type A alleles (Fig. 5C), and was particularly prevalent in Asian populations (Fig. 5D). These results suggest that, based on patterns of HLA class I diversity, populations in Asia appear to be well adapted to strong HLA class I antigen presentation of SARS-CoV-2 peptides.

**Fig. 6:**
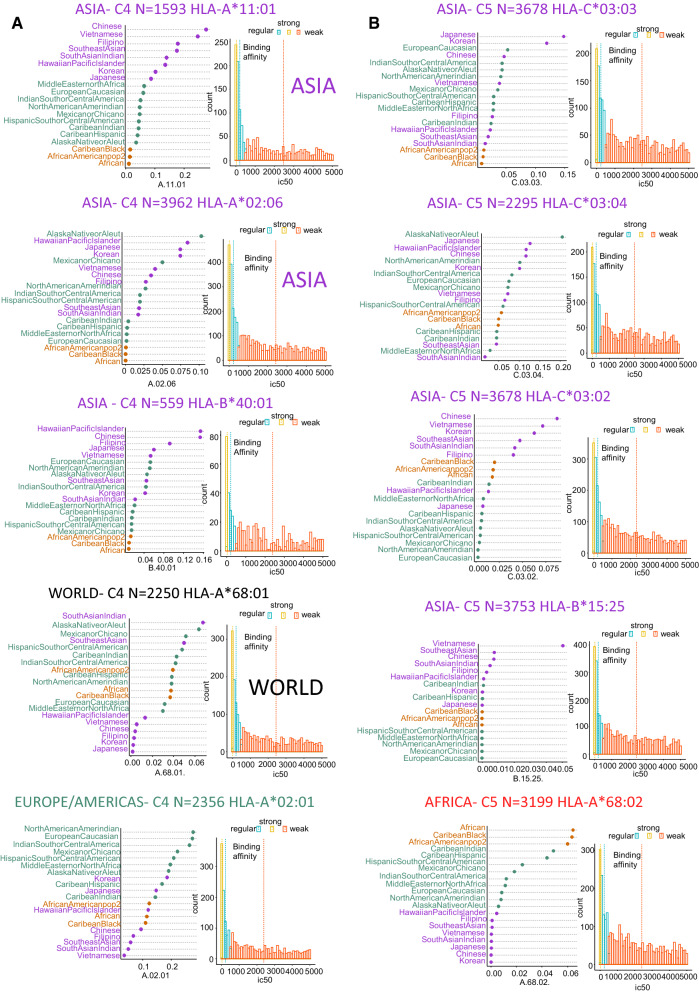
Top 10 predicted strong presenters of SARS-CoV-2 peptides among globally prevalent MHC class-I alleles. **A** Global prevalence and immunopeptidome-binding affinity of strongly presenting HLA alleles found in cluster C4; **B** Global prevalence and immunopeptidome-binding affinity of strongly presenting HLA alleles found in cluster C5

Of the 10 strongest-binding HLA alleles, seven were predicted to be highly prevalent in Asia (Fig. 6A, B): HLA-A*11:01, predicted to bind 1593 peptides and with a prevalence > 0.020 in Chinese and Vietnamese populations (Fig. 6A); HLA-A*02:06, 3962 peptides, prevalence > 0.075 in Hawaiian, Japanese, and Korean populations (Fig. 6A); HLA-B*40:01, 559 peptides, prevalence > 0.16 in Hawaiian and Chinese populations (Fig. 6A); HLA-C*03:03, 3678 peptides, prevalence > 0.10 in Japanese and Korean populations (Fig. 6B); HLA-C*03:04, 2295 peptides, prevalence > 0.10 in Japanese, Hawaiian, and Chinese populations (Fig. 6B); HLA-C*03:02, 3678 peptides, prevalence > 0.075 in the Chinese population (Fig. 6B); and HLA-B*15:25, 3753 peptides, prevalence >  = 0.05 in the Vietnamese population (Fig. 6B). Of the remaining three strong-binding alleles, one was most common in Europe/Americas (HLA-A*02:01, predicted to bind 2356 peptides, prevalence > 0.20 in American and Caucasian populations; Fig. 6A) and another was most common in African populations (HLA-A*68:02, predicted to bind 3199 peptides, prevalence > 0.06 in African populations; Fig. 6B).

**Fig. 7:**
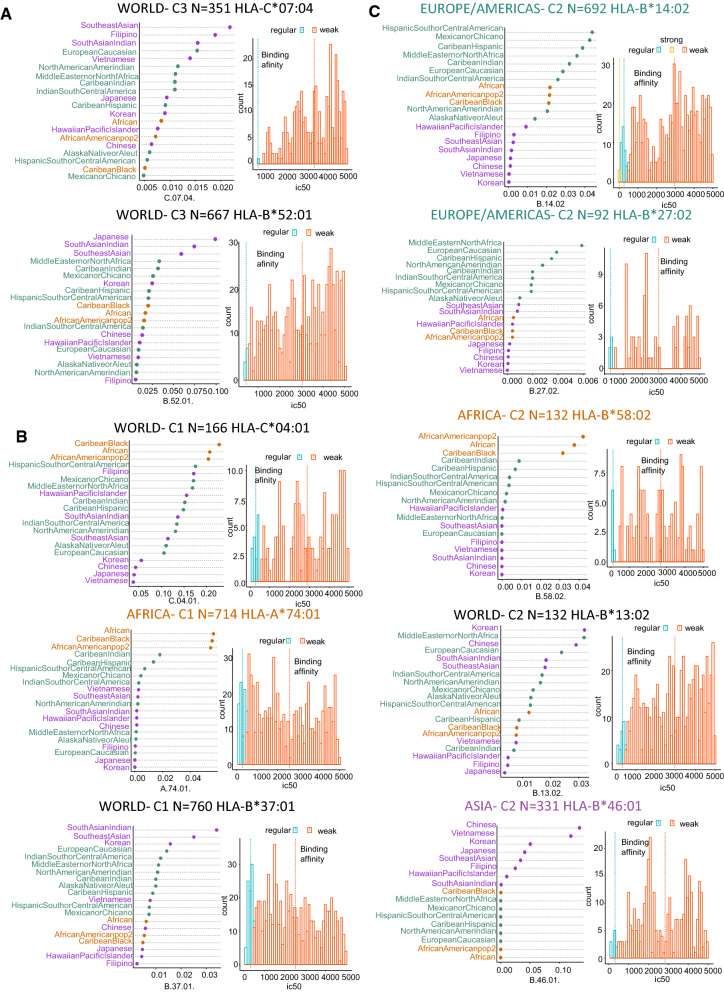
Top 10 predicted weak presenters of SARS-CoV-2 peptides among globally prevalent MHC class-I alleles. **A** Global prevalence and immunopeptidome-binding affinity of weakly presenting HLA alleles found in cluster C3. **B** Global prevalence and immunopeptidome-binding affinity of weakly presenting HLA alleles found in cluster C1. **C** Global prevalence and immunopeptidome-binding affinity of weakly presenting HLA alleles found in cluster C2

With respect to the 10 poorest-presenting HLA alleles (Fig. 7), five had worldwide distributions: HLA-C*07:04, predicted to bind 351 peptides (Fig. 7A); HLA-B*52:01, 667 peptides (Fig. 7A); HLA-C*04:01, 166 peptides (Fig. 7B); HLA-B*37:01, 760 peptides (Fig. 7B); and HLA-B*46:01, 331 peptides (Fig. 7C). Two were found with higher prevalence in African populations—HLA-A*74:01, binding 714 peptides (prevalence > 0.05, Fig. 7B); and HLA-B*58:02, binding 132 peptides (prevalence > 0.03, Fig. 7C)—and one of the weakest-presenting globally distributed alleles was also particularly frequent in African populations (HLA-C*04:01, 166 peptides, prevalence > 0.20; Fig. 7B). In Western populations, two weakly presenting HLA alleles were common: HLA-B*14:02, binding 692 peptides, prevalence > 0.05 particularly in Hispanic populations (Fig. 7C); and HLA-B*27:02, which was predicted to bind only 92 peptides (prevalence > 0.002, Fig. 7C). In Asian populations, only one weak-binding allele was identified: HLA-B*46:01, binding 331 peptides, prevalence > 0.10 in Chinese and Vietnamese populations (Fig. 7C). Based on these results, it appears that Asian populations may harbor a higher proportion of HLA class I alleles that are able to strongly present peptides of SARS-CoV-2.

## Discussion

Infection by the SARS-CoV-2 virus can provoke acute respiratory symptoms that may result in admission to an intensive care unit and/or death. During SARS-CoV-2 pathogenesis, the infiltration of pro-inflammatory monocytes and macrophages has been identified as a key mediating factor in both the hyper-inflammatory response produced during viral shedding in the infection phase and in the cytokine storm associated with severe cases [5, 23]. The activation and differentiation of monocytes into macrophages has been linked to the activity of IL6 and GM-CSF cytokines, which appear to play an important role in the cytokine storm of COVID-19 patients admitted to intensive care units [39]. The role of monocytes in enhancing proinflammatory signaling and the antiviral response is not unique to SARS-CoV-2; similar effects have been reported for influenza, herpes, and Zika viruses [40]. In this work, we observed a clear correlation between the antigen-presenting activity of MHC class I molecules and the viral load of SARS-CoV-2 in the lung tissue of patients. Patients with high viral loads harbored significant illness duration and time from admission to death. These patients tend to have positive relation with hypotension at admission and negative relation with hypertension as preexisting disease suggesting a relation between tension regulation and the level of SARS-COV2 in lungs. Recently a meta-analysis showed an association between Renin–Angiotensin–Aldosterone System inhibitors and clinical outcomes in patients with COVID-19 [41]. Activation of MHC class I antigen processing and presentation during the innate immune response implicated all components of the proteasome, but a particularly pronounced correlation was detected between levels of SARS-CoV-2 RNA and activation of components of the immunoproteasome. Immunoproteasomes are formed by replacing the standard proteasome subunits β1c, β2c, and β5c with the immunoproteasome subunits LMP2 (alias PSMB9), LMP7 (alias PSMB8), and MECL-1 (alias PSMB10); this occurs principally in cells of hematopoietic origin after stimulation with the pro-inflammatory cytokine IFN-γ [13, 42]. Regulation of immunoproteasome components is accomplished via activation of a 40-kb genomic region on chromosome 6p21.32, in the MHC class II cluster. Here, this immunoproteasome activation was confirmed at the single-cell level in alveolar macrophages of patients with mild COVID-19. In the lungs, the arrival of a pathogen may provoke an innate immune response of the parenchyma that is characterized by the differentiation of bone marrow–derived monocytes into alveolar macrophages, which serve as the first line of defense against invading pathogens [43]. The immunoproteasome appears to play an important role in this process, as immunoproteasome inhibitors have been shown to be very effective in altering alveolar macrophage polarization [14]. In analyzing single-cell transcriptomes of bronchoalveolar lavages from patients with mild and severe COVID-19, we detected the activation of the immunoproteasome component PSMB8 in the CD14+/CD16+ cell subpopulation, but these expression patterns were more muted in patients with severe COVID-19. In mild cases of COVID-19, we further observed that the expression of PSMB8 in alveolar monocytes/macrophages was correlated with that of genes associated with the polarization of M1 macrophages, such as CD68, MYD88, and STAT1 [33]. When we constructed a single-cell trajectory for the activation of the immunoproteasome (i.e. expression of PSMB8) in polarized M1 alveolar macrophages, we detected the involvement of lipid catabolic processes and lipid transport in cells from patients with mild COVID-19, but this functionality appeared to be impaired in equivalent cells from patients with severe COVID-19. This finding was consistent with the fact that alveolar macrophages have been found to differentially express genes that are involved in lipid metabolism compared with other populations of macrophages [44]. Likewise, these lipid-related modifications are in agreement with our previous finding that in patients with severe COVID-19, alveolar monocytes/macrophages demonstrate repression of PPARγ, a key regulator of lipid storage and metabolism [45]. In GM-CSF-deficient mice, the resulting repression of PPARγ in alveolar macrophages results in the accumulation of lipids; the overexpression of PPARγ restores the ability of these animals to clear alveolar lipids [46]. Our cell trajectory also highlighted a difference in the activation of calreticulin in equivalent cell populations in mild versus severe cases of COVID-19, with the latter demonstrating relative impairment. Calreticulin plays an accessory role in immunoproteasome activity through its function as a chaperone in the endoplasmic reticulum [28], but mutations in this gene are frequently implicated in myeloproliferative disorders such as essential thrombocythemia or older patients with primary myelofibrosis [47]. The PSMB8 cell trajectory highlighted a similar pattern for MARCO (macrophage receptor with collagenous structure) in M1-polarized alveolar macrophages: activation in mild COVID-19 cases and impairment in severe COVID-19 cases. MARCO is a scavenger receptor that has been implicated in the clearance of apoptotic cells and in the resolution of long-term lung inflammation; MARCO-deficient mice show early, enhanced development of inflammation in response to infection by influenza [48].

Viral infections provoke proinflammatory signaling and antiviral responses in and by monocytes/macrophages [40]. In human alveolar macrophages, one part of this response involves antigen presentation by immunoproteasome-specific MHC class I molecules [49], which forms the basis for T cell–mediated immunity. An improved understanding of this process in COVID-19 is important for the optimization of therapeutic approaches, which to date have suffered from a lack of knowledge on how heterogeneity in the immune response is linked to disease severity. Antigen presentation is carried out by HLA molecules, which present a rich and complex repertoire of peptides that inform T cells about abnormalities in the genome, transcriptome, and proteome of infected or malignant cells [50, 51]; together, this set of peptides is known as the HLA peptidome. Here, we investigated this peptidome in the context of SARS-CoV-2 and the 69 most prevalent alleles of HLA (A, B, and C types) worldwide. Our predictions of the interactions between these alleles and viral peptides reveal a high diversity of MHC class I antigen presentation. Among the alleles that were predicted to bind SARS-CoV-2 peptides most strongly, we found an overrepresentation of HLA-A alleles, which could be explained by the fact that this allele type is more diverse in the human population than types B and C [52]. Among the 10 strongest binders of peptides, we found two alleles (HLA-A*68:01 and HLA-B*15:25, Fig. 6) that had been previously identified in an independent study as the best binders for respiratory viruses, including coronaviruses [38]. In our study, the HLA-A*02:01 allele was the only strong binder in Western populations, especially in European and American populations (Fig. 6A); interestingly, it was experimentally confirmed that this allele could induce a SARS-CoV-2–specific CD8+ T cell response [53]. In general, our results are concordant with previous work, which supports the validity of our approach. For example, Nguyen et al. [54] conducted an elaborate in silico analysis of the binding affinities between SARS-CoV-2 peptides and various MHC class I molecules, representing 145 HLA-A, B, and C genotypes. Like us, they also predicted that the HLA-B*46:01 allele bound to a fewer number of viral peptide antigens and as a result postulated that individuals with this allele have an increased risk of vulnerability to SARS-CoV-2 infection [54]. HLA-B*46:01 is poorly expressed on the cell surface and has a low-diversity peptidome, possibly due to its prolonged association with HLA-specific chaperones and intracellular retention [55]. Of the 10 weakest-binding alleles highlighted by our study, five were also identified in a study by La Porta and Zapperi [56] that was performed with version 4.0 of the netMHCpan algorithm [57]. This bioinformatics analysis of binding between MHC class I and SARS-CoV-2 focused only on structural proteins (S,N,E,M) of the virus and two of the strong-binding HLA alleles that in our study were common to the Asian population: HLA-A*11:01 and HLA-B*40:01. In our work, instead, we examined MHC class I binding affinity to the totality of the SARS-CoV-2 proteome (38 proteins), including nonstructural proteins (NSP). Two-thirds of the viral RNA of SARS-CoV-2 is found within the first ORF (ORF1a/b), where translation of the two polyproteins pp1a and pp1ab occurs, together with that of 16 non-structural proteins [58]. Previous work on the NSP proteins of this virus has recognized their influence in infection. Within the sequence of non-structural protein 2 (NSP2), positive selection pressure facilitated a mutation at amino-acid position 321, from a non-polar amino acid (in the bat SARS-like coronavirus) to glutamine. This amino-acid substitution conferred the ability to form stable hydrogen bonds with the endosome-associated protein, and a resulting enhancement in viral pathogenesis [59]. Similarly, the new (L) and ancestral (S) lineages of SARS-CoV-2 differ in a mutation in amino acid 84 of ORF8, with the result that the L lineage has gradually become much more prevalent [60]. By predicting the immunopeptidome from the entire SARS-CoV-2 proteome, we aimed to take into account the influence of these nonstructural viral proteins on the immune response.

Our study highlighted one strongly binding allele, HLA-A*68:02, that has a prevalence of more than 0.06 in African and African-American populations, as well as three weakly binding alleles—HLA-A*74:01, HLA-B*58:02, and HLA-C*04:01—that were highly prevalent in these populations. Unfortunately, these observations do not shed any light on the epidemiological paradox that the spread of COVID-19 in Africa has been relatively restrained, while the American Black population has been more severely affected [61]. In our study, we observed that the highest prevalence of strongly binding HLA class I alleles, with affinity for the entire proteome of SARS-CoV-2, was in Asian populations, which suggests that antigen presentation in these populations may be more efficient. Of particular interest were the alleles HLA-A*11:01, which had a prevalence higher than 0.2 in Chinese and Vietnamese populations; HLA-A*02:06, which had a prevalence higher than 0.07 in Hawaiian (Pacific Islander), Korean, and Japanese populations; HLA-B*40:01, with a prevalence higher than 0.16 in Hawaiian (Pacific Islander) and Chinese populations; HLA-C*03:03, with a prevalence higher than 0.10 in Japanese and Korean populations; HLA-C03:04, with a prevalence higher than 0.10 in Hawaiian (Pacific Islander) and Japanese populations; HLA-C*03:02 with a prevalence higher than 0.06 in Chinese, Vietnamese, and Korean populations; and HLA-B*15:25, with a prevalence higher than 0.05 in the Vietnamese population. As a result of this allelic diversity in HLA class I antigen presenters, Asian populations may present more strong binders for SARS-CoV-2 than populations in other parts of the world, which could potentially be a contributing factor in why the COVID-19 pandemic is now less severe in that region.

## Conclusions

We observed that MHC class I antigen presentation is strongly activated in the lung tissue of COVID-19 patients, and this activation, specifically via the expression of a genetic cluster of immunoproteasome components, is correlated with the amount of SARS-CoV-2 RNA present in these tissues. By investigating the PSMB8 subunit of the immunoproteasome, we identified single-cell heterogeneity in the expression of this gene that revealed a cell trajectory for alveolar M1 macrophages during mild stages of COVID-19. Specifically, patterns of single-cell heterogeneity in bronchoalveolar cells highlighted an impairment in alveolar clearance mechanisms (e.g., lipid transport and catabolism, MARCO scavenger receptor for apoptotic cells) in equivalent cells of patients with severe COVID-19. Finally, predictions of binding affinity for the SARS-CoV-2 immunopeptidome among different human populations identified high diversity in Asian populations for strong MHC class-I presenters of the SARS-CoV-2 virus.

## Supplementary Information

**Additional file 1: Figure S1.** Bronchoalveolar single-cell expression of immunoproteasome-related markers in COVID-19 patients. **Figure S2.** Bronchoalveolar single-cell expression of CD163 and its correlation with the expression of immunoproteasome-related markers in COVID-19 patients. **Figure S3.** Unsupervised clustering of 20 ethnic groups into larger world regions (Africa, Asia, and the Europe/America world) based of the prevalence of 69 HLA-A,B,C alleles. **Figure S4.** Random Forest validation of population clustering in world regions (Africa, Asia, and Europe/Americas, world) based on the prevalence of 69 HLA-A,B,C alleles. **Table S1.** Table of univariate analysis of admission symptoms for COVID-19 patients in the transcriptome GSE150316 cohort according to their stratification with low and high levels of SARS-COV2 detected in lung tissue; **Table S2.** Table of univariate analysis of preexisting disease for COVID-19 patients in the transcriptome GSE150316 cohort according to their stratification with low and high levels of SARS-COV2 detected in lung tissue; **Table S3.** Table of univariate analysis for treatment intervention for COVID-19 patients in the transcriptome GSE150316 cohort according to their stratification with low and high levels of SARS-COV2 detected in lung tissue; **Table S4.** Table of genes implicated in MHC class I antigen presentation whose upregulation in the lungs of COVID-19 patients reflected the amount of SARS-CoV-2 virus detected in the tissue. **Table S5.** Genes identified as significant in the PSMB8-defined cell trajectory of CD14+/CD68+ bronchoalveolar cells from COVID-19 patients. **Table S6**. Prevalence of the 69 most-frequent HLA-A,B,C alleles in 20 ethnicities with a worldwide distribution (USA NMDP bone marrow registry). **Table S7.** List of SARS-CoV-2 protein sequences used for immunoinformatics predictions of MHC class I binding. **Table S8.** Predicted MHC class I binding events between the SARS-CoV-2 immunopeptidome and 69 most prevalent alleles of HLA-A,B,C. **Table S9.** The number of 9-mer peptides bound by each HLA allele, grouped by the strength of binding.

## Data Availability

We obtained transcriptomes from COVID-19 lung biopsies from dataset GSE150316 in the Gene Expression Omnibus database (https://www.medrxiv.org/content/10.1101/2020.07.30.20165241v1). Single-cell transcriptomes from bronchoalveolar fluid lavage were obtained from dataset GSE145926 [23]. Mendeley Dataset associated with this manuscript: Supplemental table 8 database is provided as a Mendeley dataset: https://doi.org/10.17632/rjmyz3j52y.2 Internet links: https://data.mendeley.com/datasets/rjmyz3j52y/2

## Materials availability

This study did not generate specific reagents.

## Code availability

The R code for bioinformatics analyses is detailed in the Methods section of the Supplemental Information and also provided at the following address: https://github.com/cdesterke/covid19if.
