# Supplementary material for: HLA-dependent heterogeneity and macrophage immunoproteasome activation during lung COVID-19 disease
Source: J Transl Med. 2021 Jul 5;19:290. doi: 10.1186/s12967-021-02965-5 (PMC8256232; doi:10.1186/s12967-021-02965-5)
Supplement: Supplementary file 1 — Additional file 1: Figure S1. Bronchoalveolar single-cell expression of immunoproteasome-related markers in COVID-19 patients. Figure S2. Bronchoalveolar single-cell expression of CD163 and its correlation with the expression of immunoproteasome-related markers in COVID-19 patients. Figure S3. Unsupervised clustering of 20 ethnic groups into larger world regions (Africa, Asia, and the Europe/America world) based of the prevalence of 69 HLA-A,B,C alleles. Figure S4. Random Forest validation of population clustering in world regions (Africa, Asia, and Europe/Americas, world) based on the prevalence of 69 HLA-A,B,C alleles. Table S1. Table of univariate analysis of admission symptoms for COVID-19 patients in the transcriptome GSE150316 cohort according to their stratification with low and high levels of SARS-COV2 detected in lung tissue; Table S2. Table of univariate analysis of preexisting disease for COVID-19 patients in the transcriptome GSE150316 cohort according to their stratification with low and high levels of SARS-COV2 detected in lung tissue; Table S3. Table of univariate analysis for treatment intervention for COVID-19 patients in the transcriptome GSE150316 cohort according to their stratification with low and high levels of SARS-COV2 detected in lung tissue; Table S4. Table of genes implicated in MHC class I antigen presentation whose upregulation in the lungs of COVID-19 patients reflected the amount of SARS-CoV-2 virus detected in the tissue. Table S5. Genes identified as significant in the PSMB8-defined cell trajectory of CD14+/CD68+ bronchoalveolar cells from COVID-19 patients. Table S6. Prevalence of the 69 most-frequent HLA-A,B,C alleles in 20 ethnicities with a worldwide distribution (USA NMDP bone marrow registry). Table S7. List of SARS-CoV-2 protein sequences used for immunoinformatics predictions of MHC class I binding. Table S8. Predicted MHC class I binding events between the SARS-CoV-2 immunopeptidome and 69 most prevalent alleles of HLA-A,B, [file 12967_2021_2965_MOESM1_ESM.docx]

**Supplemental Information**

***Supplemental material***

**Supplemental figures: Supplemental Figure 1: Bronchoalveolar single-cell expression of immunoproteasome-related markers in COVID-19 patients. Supplemental Figure 2: Bronchoalveolar single-cell expression of CD163 and its correlation with the expression of immunoproteasome-related markers in COVID-19 patients. Supplemental Figure 3: Unsupervised clustering of 20 ethnic groups into larger world regions (Africa, Asia, and the Europe/Americs world) based of the prevalence of 69 HLA-A,B,C alleles. Supplemental Figure 4: Random Forest validation of population clustering in world regions (Africa, Asia, and Europe/Americas, world) based on the prevalence of 69 HLA-A,B,C alleles.**

**Supplemental tables: Supplemental Table 1: Table of univariate analysis for admission symptoms for COVID-19 patients in transcriptome GSE150316 cohort according their stratification with low and high level of SARS-COV2 detected in lung tissue; Supplemental Table 2: Table of univariate analysis for preexisting disease for COVID-19 patients in transcriptome GSE150316 cohort according their stratification with low and high level of SARS-COV2 detected in lung tissue; Supplemental Table 3: Table of univariate analysis for treatment intervention for COVID-19 patients in transcriptome GSE150316 cohort according their stratification with low and high level of SARS-COV2 detected in lung tissue; Supplemental Table 4: Table of genes implicated in MHC class I antigen presentation whose upregulation in the lungs of COVID-19 patients reflected the amount of SARS-CoV2 virus detected in the tissue. Supplemental Table 5: Genes identified as significant in the PSMB8-defined cell trajectory of CD14+/CD68+ bronchoalveolar cells from COVID-19 patients. Supplemental Table 6: Prevalence of the 69 most-frequent HLA-A,B,C alleles in 20 ethnicities with a worldwide distribution (USA NMDP bone marrow registry). Supplemental Table 7: List of SARS-CoV2 protein sequences used for immunoinformatics predictions of MHC class I binding. Supplemental Table 8: Predicted MHC class I binding events between the SARS-CoV2 immunopeptidome and 69 most prevalent alleles of HLA-A,B,C. Supplemental Table 9: The number of 9-mer peptides bound by each HLA allele, grouped by the strength of binding.**

**As Supplemental table 8 is a database and have a very big size, it is provided as Mendeley dataset: DOI: 10.17632/rjmyz3j52y.2**

Supplemental Table 8 link : <https://data.mendeley.com/datasets/rjmyz3j52y/2>

**Supplemental Figure 1: Bronchoalveolar single-cell expression of immunoproteasome-related markers in COVID-19 patients.**

**Supplemental Figure 2: Bronchoalveolar single-cell expression of CD163 and its correlation with the expression of immunoproteasome-related markers in COVID-19 patients:** HD: healthy donor, MC: mild COVID19, SC: severe COVID19, A/ single cell expression of CD163 in human bronchalveolar fluid lavages, Boxplot and UMAP dimension reduction; B/ biplot of CD163 single cell expression versus immunoproteasome components in human bronchoalveolar fluid lavages

**Supplemental Figure 3: Unsupervised clustering of 20 ethnic groups into larger world regions (Africa, Asia, and the Europe/Americs world) based of the prevalence of 69 HLA-A,B,C alleles.** A/principal component analysis of 69 MHC class I more prevalent in the world, color stratification could be done with 3 world regions: Africa, Asia and Europe/America; B/Correlation plot of the 69 MHC I alleles used during principal component analysis; C/Dotchart of MHC class I prevalences at worldwide level for three example of discriminant allele, respective discrimination for: Asia region (HLA-C*08:01), Europe/Americas region (HLA-A*25:01); Africa region (HLA-B*53:01).

**Supplemental Figure 4: Random Forest validation of population clustering in world regions (Africa, Asia, and Europe/Americas, world) based on the prevalence of 69 HLA-A,B,C alleles.** A/Machine learning random forest error rate plot for discrimination of the 3 world region, Africa, Asia, Europe/America based on 69 more prevalent MHC I alleles; B/ Misclassification table during Random Forest learning; C/ heatmap of classification matrix after Random Forest learning; D/ Multidimensionnal scaling plot of world region attribution based on MHC I allele frequencies; E/ World region importance during machine learning to class region with MHC I allele frequencies: on left (mean decrease accuracy) and on the right (mean decrease gini)

**Supplemental Table 1: Table of univariate analysis for admission symptoms for COVID-19 patients in transcriptome GSE150316 cohort according their stratification with low and high level of SARS-COV2 detected in lung tissue:** in yellow: significant parameters, in green: parameters harboring a tendency for a negative association with high level of SARS-COV2, in pink: parameters harboring a tendency for a positive association with high level of SARS-COV2; quantitative parameters: mean (sd), qualitative parameters: numbers (percentages).

| **Variable** | **Level** | **group_HIGH = 0 (n=6)** | **group_HIGH = 1 (n=8)** | **Total (n=14)** | **p-value** |
| --- | --- | --- | --- | --- | --- |
| Hospital.Center | Columbia Univ Medical Center | 1 (16.7) | 2 (25.0) | 3 (21.4) |  |
|  | Massachusetts General Center | 5 (83.3) | 6 (75.0) | 11 (78.6) | 1.0000000 |
| SARS.COV2.RNA.ISH.lung.PMID..33298930 | mean (sd) | 0.7 (1.2) | 45.6 (32) | 26.4 (32.9) | 0.0006677 |
| age | mean (sd) | 48.3 (16) | 62.5 (15.8) | 56.4 (16.9) | 0.0989656 |
| gender.F | no | 3 (50.0) | 3 (37.5) | 6 (42.9) |  |
|  | YES | 3 (50.0) | 5 (62.5) | 8 (57.1) | 1.0000000 |
| PRESsymp_Fever | no | 1 (16.7) | 3 (50.0) | 4 (33.3) |  |
|  | YES | 5 (83.3) | 3 (50.0) | 8 (66.7) | 0.5402914 |
|  | missing | 0 | 2 | 2 |  |
| PRESsymp_Fever.with.chill | no | 5 (83.3) | 6 (100.0) | 11 (91.7) |  |
|  | YES | 1 (16.7) | 0 (0.0) | 1 (8.3) | 1.0000000 |
|  | missing | 0 | 2 | 2 |  |
| PRESsymp_cough | no | 1 (16.7) | 5 (83.3) | 6 (50.0) |  |
|  | YES | 5 (83.3) | 1 (16.7) | 6 (50.0) | 0.0832645 |
|  | missing | 0 | 2 | 2 |  |
| PRESsymp_hypoxemia | no | 1 (16.7) | 4 (66.7) | 5 (41.7) |  |
|  | YES | 5 (83.3) | 2 (33.3) | 7 (58.3) | 0.2415666 |
|  | missing | 0 | 2 | 2 |  |
| PRESsymp_homeless | no | 5 (83.3) | 6 (100.0) | 11 (91.7) |  |
|  | YES | 1 (16.7) | 0 (0.0) | 1 (8.3) | 1.0000000 |
|  | missing | 0 | 2 | 2 |  |
| PRESsymp_myalgia | no | 3 (50.0) | 6 (100.0) | 9 (75.0) |  |
|  | YES | 3 (50.0) | 0 (0.0) | 3 (25.0) | 0.1824224 |
|  | missing | 0 | 2 | 2 |  |
| PRESsymp_sore.throat | no | 5 (83.3) | 6 (100.0) | 11 (91.7) |  |
|  | YES | 1 (16.7) | 0 (0.0) | 1 (8.3) | 1.0000000 |
|  | missing | 0 | 2 | 2 |  |
| PRESsymp_headache | no | 5 (83.3) | 6 (100.0) | 11 (91.7) |  |
|  | YES | 1 (16.7) | 0 (0.0) | 1 (8.3) | 1.0000000 |
|  | missing | 0 | 2 | 2 |  |
| PRESsymp_nausea | no | 5 (83.3) | 5 (83.3) | 10 (83.3) |  |
|  | YES | 1 (16.7) | 1 (16.7) | 2 (16.7) | 1.0000000 |
|  | missing | 0 | 2 | 2 |  |
| PRESsymp_ulcer | no | 5 (83.3) | 6 (100.0) | 11 (91.7) |  |
|  | YES | 1 (16.7) | 0 (0.0) | 1 (8.3) | 1.0000000 |
|  | missing | 0 | 2 | 2 |  |
| PRESsymp_osteomyelitis | no | 5 (83.3) | 6 (100.0) | 11 (91.7) |  |
|  | YES | 1 (16.7) | 0 (0.0) | 1 (8.3) | 1.0000000 |
|  | missing | 0 | 2 | 2 |  |
| PRESsymp_fatigue | no | 6 (100.0) | 5 (83.3) | 11 (91.7) |  |
|  | YES | 0 (0.0) | 1 (16.7) | 1 (8.3) | 1.0000000 |
|  | missing | 0 | 2 | 2 |  |
| PRESsymp_dyspnea | no | 6 (100.0) | 5 (83.3) | 11 (91.7) |  |
|  | YES | 0 (0.0) | 1 (16.7) | 1 (8.3) | 1.0000000 |
|  | missing | 0 | 2 | 2 |  |
| PRESsymp_lethargy | no | 6 (100.0) | 3 (50.0) | 9 (75.0) |  |
|  | YES | 0 (0.0) | 3 (50.0) | 3 (25.0) | 0.1824224 |
|  | missing | 0 | 2 | 2 |  |
| PRESsymp_unresponsive | no | 6 (100.0) | 5 (83.3) | 11 (91.7) |  |
|  | YES | 0 (0.0) | 1 (16.7) | 1 (8.3) | 1.0000000 |
|  | missing | 0 | 2 | 2 |  |
| PRESsymp_tachycardia | no | 6 (100.0) | 5 (83.3) | 11 (91.7) |  |
|  | YES | 0 (0.0) | 1 (16.7) | 1 (8.3) | 1.0000000 |
|  | missing | 0 | 2 | 2 |  |
| PRESsymp_hypotension | no | 6 (100.0) | 4 (66.7) | 10 (83.3) |  |
|  | YES | 0 (0.0) | 2 (33.3) | 2 (16.7) | 0.4385780 |
|  | missing | 0 | 2 | 2 |  |
| PRESsymp_weakness | no | 6 (100.0) | 4 (66.7) | 10 (83.3) |  |
|  | YES | 0 (0.0) | 2 (33.3) | 2 (16.7) | 0.4385780 |
|  | missing | 0 | 2 | 2 |  |
| PRESsymp_rhinorrhea | no | 6 (100.0) | 5 (83.3) | 11 (91.7) |  |
|  | YES | 0 (0.0) | 1 (16.7) | 1 (8.3) | 1.0000000 |
|  | missing | 0 | 2 | 2 |  |
| PRESsymp_ambulance.intubated | no | 6 (100.0) | 6 (85.7) | 12 (92.3) |  |
|  | YES | 0 (0.0) | 1 (14.3) | 1 (7.7) | 1.0000000 |
|  | missing | 0 | 1 | 1 |  |
| PRESsymp_diarrhea | no | 6 (100.0) | 5 (83.3) | 11 (91.7) |  |
|  | YES | 0 (0.0) | 1 (16.7) | 1 (8.3) | 1.0000000 |
|  | missing | 0 | 2 | 2 |  |
| PRESsymp_abdominal.pain | no | 6 (100.0) | 5 (83.3) | 11 (91.7) |  |
|  | YES | 0 (0.0) | 1 (16.7) | 1 (8.3) | 1.0000000 |
|  | missing | 0 | 2 | 2 |  |
| PRESsymp_SOB.shortness.of.breath | no | 6 (100.0) | 7 (87.5) | 13 (92.9) |  |
|  | YES | 0 (0.0) | 1 (12.5) | 1 (7.1) | 1.0000000 |

**Supplemental Table 2: Table of univariate analysis for preexisting disease for COVID-19 patients in transcriptome GSE150316 cohort according their stratification with low and high level of SARS-COV2 detected in lung tissue:** in yellow: significant parameters, in green: parameters harboring a tendency for a negative association with high level of SARS-COV2, in pink: parameters harboring a tendency for a positive association with high level of SARS-COV2; quantitative parameters: mean (sd), qualitative parameters: numbers (percentages).

| **Variable** | **Level** | **group_HIGH = 0 (n=6)** | **group_HIGH = 1 (n=8)** | **Total (n=14)** | **p-value** |
| --- | --- | --- | --- | --- | --- |
| Hospital_Center | Massachusetts_General_Center | 5 (83.3) | 6 (75.0) | 11 (78.6) |  |
|  | Columbia_Univ_Medical_Center | 1 (16.7) | 2 (25.0) | 3 (21.4) | 1.0000000 |
| SARS.COV2_RNA.ISH_lung_PMID.33298930 | mean (sd) | 0.7 (1.2) | 45.6 (32) | 26.4 (32.9) | 0.0006677 |
| age | mean (sd) | 48.3 (16) | 62.5 (15.8) | 56.4 (16.9) | 0.0989656 |
| gender.F | YES | 3 (50.0) | 5 (62.5) | 8 (57.1) |  |
|  | no | 3 (50.0) | 3 (37.5) | 6 (42.9) | 1.0000000 |
| PREEXISTdisease_HTN_hypertension | YES | 3 (50.0) | 7 (87.5) | 10 (71.4) |  |
|  | no | 3 (50.0) | 1 (12.5) | 4 (28.6) | 0.3475748 |
| PREEXISTdisease_COPD_chronic_obstructivepulmonary_disease | no | 6 (100.0) | 7 (87.5) | 13 (92.9) |  |
|  | YES | 0 (0.0) | 1 (12.5) | 1 (7.1) | 1.0000000 |
| PREEXISTdisease_OSA_obstructive_sleep_apnea | YES | 1 (16.7) | 0 (0.0) | 1 (7.1) |  |
|  | no | 5 (83.3) | 8 (100.0) | 13 (92.9) | 0.8809333 |
| PREEXISTdisease_obese | YES | 2 (33.3) | 1 (12.5) | 3 (21.4) |  |
|  | no | 4 (66.7) | 7 (87.5) | 11 (78.6) | 0.7779143 |
| PREEXISTdisease_Asthma | no | 5 (83.3) | 8 (100.0) | 13 (92.9) |  |
|  | YES | 1 (16.7) | 0 (0.0) | 1 (7.1) | 0.8809333 |
| PREEXISTdisease_cancer | no | 5 (83.3) | 6 (75.0) | 11 (78.6) |  |
|  | YES | 1 (16.7) | 2 (25.0) | 3 (21.4) | 1.0000000 |
| PREEXISTdisease_metastatic_cancer | no | 5 (83.3) | 7 (87.5) | 12 (85.7) |  |
|  | YES | 1 (16.7) | 1 (12.5) | 2 (14.3) | 1.0000000 |
| PREEXISTdisease_T2DM_diabete | no | 2 (33.3) | 4 (50.0) | 6 (42.9) |  |
|  | YES | 4 (66.7) | 4 (50.0) | 8 (57.1) | 0.9378669 |
| PREEXISTdisease_NAFLD | no | 5 (83.3) | 8 (100.0) | 13 (92.9) |  |
|  | YES | 1 (16.7) | 0 (0.0) | 1 (7.1) | 0.8809333 |
| PREEXISTdisease_hypothyroidism | no | 4 (66.7) | 7 (87.5) | 11 (78.6) |  |
|  | YES | 2 (33.3) | 1 (12.5) | 3 (21.4) | 0.7779143 |
| PREEXISTdisease_sciatica | no | 5 (83.3) | 8 (100.0) | 13 (92.9) |  |
|  | YES | 1 (16.7) | 0 (0.0) | 1 (7.1) | 0.8809333 |
| PREEXISTdisease_LatentTuberculosis | no | 5 (83.3) | 8 (100.0) | 13 (92.9) |  |
|  | YES | 1 (16.7) | 0 (0.0) | 1 (7.1) | 0.8809333 |
| PREEXISTdisease_RenalTransplant | no | 5 (83.3) | 8 (100.0) | 13 (92.9) |  |
|  | YES | 1 (16.7) | 0 (0.0) | 1 (7.1) | 0.8809333 |
| PREEXISTdisease_HLD_hyperlipidemia | no | 5 (83.3) | 6 (75.0) | 11 (78.6) |  |
|  | YES | 1 (16.7) | 2 (25.0) | 3 (21.4) | 1.0000000 |
| PREEXISTdisease_CAD_coronary_artery_disease | no | 5 (83.3) | 8 (100.0) | 13 (92.9) |  |
|  | YES | 1 (16.7) | 0 (0.0) | 1 (7.1) | 0.8809333 |
| PREEXISTdisease_CHF_congestive_heart_failure | no | 5 (83.3) | 8 (100.0) | 13 (92.9) |  |
|  | YES | 1 (16.7) | 0 (0.0) | 1 (7.1) | 0.8809333 |
| PREEXISTdisease_chronicDVT_deep_vein_thrombosis | no | 5 (83.3) | 8 (100.0) | 13 (92.9) |  |
|  | YES | 1 (16.7) | 0 (0.0) | 1 (7.1) | 0.8809333 |
| PREEXISTdisease_osteomyelitis | no | 5 (83.3) | 8 (100.0) | 13 (92.9) |  |
|  | YES | 1 (16.7) | 0 (0.0) | 1 (7.1) | 0.8809333 |
| PREEXISTdisease_Lewybody_dementia | no | 6 (100.0) | 7 (87.5) | 13 (92.9) |  |
|  | YES | 0 (0.0) | 1 (12.5) | 1 (7.1) | 1.0000000 |
| PREEXISTdisease_CKD_chronic_kidney_disease | no | 6 (100.0) | 6 (75.0) | 12 (85.7) |  |
|  | YES | 0 (0.0) | 2 (25.0) | 2 (14.3) | 0.5814978 |
| PREEXISTdisease_priorCVA_cerebrovascular_accident | no | 6 (100.0) | 7 (87.5) | 13 (92.9) |  |
|  | YES | 0 (0.0) | 1 (12.5) | 1 (7.1) | 1.0000000 |
| PREEXISTdisease_Addison.s_disease | no | 6 (100.0) | 7 (87.5) | 13 (92.9) |  |
|  | YES | 0 (0.0) | 1 (12.5) | 1 (7.1) | 1.0000000 |
| PREEXISTdisease_GERD_gastrointestinal_reflex_disease | no | 6 (100.0) | 5 (62.5) | 11 (78.6) |  |
|  | YES | 0 (0.0) | 3 (37.5) | 3 (21.4) | 0.3010710 |
| PREEXISTdisease_Sarcoidose | no | 6 (100.0) | 7 (87.5) | 13 (92.9) |  |
|  | YES | 0 (0.0) | 1 (12.5) | 1 (7.1) | 1.0000000 |
| PREEXISTdisease_alzeihmer.s_disease | no | 6 (100.0) | 6 (75.0) | 12 (85.7) |  |
|  | YES | 0 (0.0) | 2 (25.0) | 2 (14.3) | 0.5814978 |
| PREEXISTdisease_dvp_delay | no | 6 (100.0) | 7 (87.5) | 13 (92.9) |  |
|  | YES | 0 (0.0) | 1 (12.5) | 1 (7.1) | 1.0000000 |
| PREEXISTdisease_autism | no | 6 (100.0) | 7 (87.5) | 13 (92.9) |  |
|  | YES | 0 (0.0) | 1 (12.5) | 1 (7.1) | 1.0000000 |
| PREEXISTdisease_epilepsy | no | 6 (100.0) | 7 (87.5) | 13 (92.9) |  |
|  | YES | 0 (0.0) | 1 (12.5) | 1 (7.1) | 1.0000000 |
| PREEXISTdisease_bipolar | no | 6 (100.0) | 7 (87.5) | 13 (92.9) |  |
|  | YES | 0 (0.0) | 1 (12.5) | 1 (7.1) | 1.0000000 |
| PREEXISTdisease_osteoarthritis | no | 6 (100.0) | 6 (75.0) | 12 (85.7) |  |
|  | YES | 0 (0.0) | 2 (25.0) | 2 (14.3) | 0.5814978 |
| PREEXISTdisease_psoriasis | no | 6 (100.0) | 7 (87.5) | 13 (92.9) |  |
|  | YES | 0 (0.0) | 1 (12.5) | 1 (7.1) | 1.0000000 |

**Supplemental Table 3: Table of univariate analysis for treatment intervention for COVID-19 patients in transcriptome GSE150316 cohort according their stratification with low and high level of SARS-COV2 detected in lung tissue:** in yellow: significant parameters, in green: parameters harboring a tendency for a negative association with high level of SARS-COV2, in pink: parameters harboring a tendency for a positive association with high level of SARS-COV2; quantitative parameters: mean (sd), qualitative parameters: numbers (percentages).

| **Variable** | **Level** | **group_HIGH = 0 (n=6)** | **group_HIGH = 1 (n=8)** | **Total (n=14)** | **p-value** |
| --- | --- | --- | --- | --- | --- |
| Hospital_Center | Massachusetts_General_Center | 5 (83.3) | 6 (75.0) | 11 (78.6) |  |
|  | Columbia_Univ_Medical_Center | 1 (16.7) | 2 (25.0) | 3 (21.4) | 1.0000000 |
| SARS.COV2_RNA.ISH_lung_PMID._33298930 | mean (sd) | 0.7 (1.2) | 45.6 (32) | 26.4 (32.9) | 0.0006677 |
| age | mean (sd) | 48.3 (16) | 62.5 (15.8) | 56.4 (16.9) | 0.0989656 |
| gender_F | YES | 3 (50.0) | 5 (62.5) | 8 (57.1) |  |
|  | no | 3 (50.0) | 3 (37.5) | 6 (42.9) | 1.0000000 |
| immune_suppression | no | 3 (60.0) | 5 (62.5) | 8 (61.5) |  |
|  | YES | 2 (40.0) | 3 (37.5) | 5 (38.5) | 1.0000000 |
|  | missing | 1 | 0 | 1 |  |
| COVID19TTT_vancomycin | no | 4 (80.0) | 6 (85.7) | 10 (83.3) |  |
|  | YES | 1 (20.0) | 1 (14.3) | 2 (16.7) | 1.0000000 |
|  | missing | 1 | 1 | 2 |  |
| COVID19TTT_cefepime | no | 4 (80.0) | 5 (71.4) | 9 (75.0) |  |
|  | YES | 1 (20.0) | 2 (28.6) | 3 (25.0) | 1.0000000 |
|  | missing | 1 | 1 | 2 |  |
| COVID19TTT_metronidazole | no | 4 (80.0) | 7 (100.0) | 11 (91.7) |  |
|  | YES | 1 (20.0) | 0 (0.0) | 1 (8.3) | 0.8598643 |
|  | missing | 1 | 1 | 2 |  |
| COVID19TTT_hydroxychloroquine | YES | 5 (100.0) | 3 (42.9) | 8 (66.7) |  |
|  | no | 0 (0.0) | 4 (57.1) | 4 (33.3) | 0.1472991 |
|  | missing | 1 | 1 | 2 |  |
| COVID19TTT_atorvastatin | YES | 4 (80.0) | 1 (14.3) | 5 (41.7) |  |
|  | no | 1 (20.0) | 6 (85.7) | 7 (58.3) | 0.0924596 |
|  | missing | 1 | 1 | 2 |  |
| COVID19TTT_ceftriaxone | YES | 3 (60.0) | 2 (28.6) | 5 (41.7) |  |
|  | no | 2 (40.0) | 5 (71.4) | 7 (58.3) | 0.6206907 |
|  | missing | 1 | 1 | 2 |  |
| COVID19TTT_azithromycin | YES | 4 (80.0) | 3 (42.9) | 7 (58.3) |  |
|  | no | 1 (20.0) | 4 (57.1) | 5 (41.7) | 0.4884223 |
|  | missing | 1 | 1 | 2 |  |
| COVID19TTT_pravastatin | no | 4 (80.0) | 7 (100.0) | 11 (91.7) |  |
|  | YES | 1 (20.0) | 0 (0.0) | 1 (8.3) | 0.8598643 |
|  | missing | 1 | 1 | 2 |  |
| illness_duration | mean (sd) | 16.6 (5.6) | 8.5 (2.6) | 13 (6) | 0.0082387 |
|  | missing | 1 | 4 | 5 |  |
| time_from_admission_to_death | mean (sd) | 14.3 (9.4) | 3.4 (2.3) | 8.5 (8.5) | 0.0029170 |
|  | missing | 0 | 1 | 1 |  |
| mechanical_ventilation | YES | 5 (100.0) | 4 (57.1) | 9 (75.0) |  |
|  | no | 0 (0.0) | 3 (42.9) | 3 (25.0) | 0.3104944 |
|  | missing | 1 | 1 | 2 |  |

**Supplemental Table 4: Table of genes implicated in MHC class I antigen presentation whose upregulation in the lungs of COVID-19 patients reflected the amount of SARS-CoV-2 virus detected in the tissue.** Transcriptome fold change in expression between COVID19 patients with high level of SARS-COV2 in the lung and those with low level of of SARS-COV2 in the lung: columns describe respective gene symbol, gene description, NCBI gene identifier and their fold change in expression

| gene symbol | description | Gene ID | fonction | Fold change LUNG HIGH/LOW SARS-COV2 |
| --- | --- | --- | --- | --- |
| TAP1 | transporter 1, ATP binding cassette subfamily B member | 6890 | Antigen processing cross presentation | 37.14 |
| TAP2 | transporter 2, ATP binding cassette subfamily B member | 6891 | Antigen processing cross presentation | 13.23 |
| PSMB8 | proteasome 20S subunit beta 8 | 5696 | Antigen processing cross presentation | 11.73 |
| HLA-C | major histocompatibility complex, class I, C | 3107 | Antigen processing cross presentation | 38.66 |
| SEC61B | SEC61 translocon subunit beta | 10952 | Antigen processing cross presentation | 2.39 |
| HLA-B | major histocompatibility complex, class I, B | 3106 | Antigen processing cross presentation | 35.12 |
| PSMA4 | proteasome 20S subunit alpha 4 | 5685 | Antigen processing cross presentation | 6.45 |
| TLR2 | toll like receptor 2 | 7097 | Antigen processing cross presentation | 6.18 |
| PSME1 | proteasome activator subunit 1 | 5720 | Antigen processing cross presentation | 13.94 |
| PSMA3 | proteasome 20S subunit alpha 3 | 5684 | Antigen processing cross presentation | 5.91 |
| PSMB9 | proteasome 20S subunit beta 9 | 5698 | Antigen processing cross presentation | 8.47 |
| HLA-A | major histocompatibility complex, class I, A | 3105 | Antigen processing cross presentation | 17.63 |
| B2M | beta-2-microglobulin | 567 | Antigen processing cross presentation | 25.24 |
| CHUK | component of inhibitor of nuclear factor kappa B kinase complex | 1147 | Antigen processing cross presentation | 2.96 |
| PSME2 | proteasome activator subunit 2 | 5721 | Antigen processing cross presentation | 4.87 |
| PDIA3 | protein disulfide isomerase family A member 3 | 2923 | Antigen processing cross presentation | 4.46 |
| SEC22B | SEC22 homolog B, vesicle trafficking protein | 9554 | Antigen processing cross presentation | 2.74 |
| CTSL | cathepsin L | 1514 | Antigen processing cross presentation | 7.85 |
| PSMA1 | proteasome 20S subunit alpha 1 | 5682 | Antigen processing cross presentation | 4.36 |
| PSMD1 | proteasome 26S subunit, non-ATPase 1 | 5707 | Antigen processing cross presentation | 3.01 |
| HLA-F | major histocompatibility complex, class I, F | 3134 | Antigen processing cross presentation | 5.08 |
| PSMA2 | proteasome 20S subunit alpha 2 | 5683 | Antigen processing cross presentation | 4.33 |
| PSMD7 | proteasome 26S subunit, non-ATPase 7 | 5713 | Antigen processing cross presentation | 2.38 |
| PSMB3 | proteasome 20S subunit beta 3 | 5691 | Antigen processing cross presentation | 2.46 |
| FCGR1A | Fc fragment of IgG receptor Ia | 2209 | Antigen processing cross presentation | 5.51 |
| PSMB1 | proteasome 20S subunit beta 1 | 5689 | Antigen processing cross presentation | 2.19 |
| PSMD14 | proteasome 26S subunit, non-ATPase 14 | 10213 | Antigen processing cross presentation | 2.33 |
| TLR4 | toll like receptor 4 | 7099 | Antigen processing cross presentation | 1.82 |
| PSMC6 | proteasome 26S subunit, ATPase 6 | 5706 | Antigen processing cross presentation | 2.16 |
| PSMD8 | proteasome 26S subunit, non-ATPase 8 | 5714 | Antigen processing cross presentation | 2.46 |
| PSMC3 | proteasome 26S subunit, ATPase 3 | 5702 | Antigen processing cross presentation | 5.25 |
| LY96 | lymphocyte antigen 96 | 23643 | Antigen processing cross presentation | 1.24 |
| HLA-E | major histocompatibility complex, class I, E | 3133 | Antigen processing cross presentation | 7.02 |
| SEC61A2 | SEC61 translocon subunit alpha 2 | 55176 | Antigen processing cross presentation | 1.97 |
| STX4 | syntaxin 4 | 6810 | Antigen processing cross presentation | 1.55 |
| PSMA5 | proteasome 20S subunit alpha 5 | 5686 | Antigen processing cross presentation | 2.49 |
| CD14 | CD14 molecule | 929 | Antigen processing cross presentation | 1.97 |
| SEC61A1 | SEC61 translocon subunit alpha 1 | 29927 | Antigen processing cross presentation | 1.79 |
| PSMC1 | proteasome 26S subunit, ATPase 1 | 5700 | Antigen processing cross presentation | 2.63 |
| IKBKG | inhibitor of nuclear factor kappa B kinase regulatory subunit gamma | 8517 | Antigen processing cross presentation | 1.45 |
| PSMD2 | proteasome 26S subunit ubiquitin receptor, non-ATPase 2 | 5708 | Antigen processing cross presentation | 1.46 |
| ITGB5 | integrin subunit beta 5 | 3693 | Antigen processing cross presentation | 1.31 |
| HLA-G | major histocompatibility complex, class I, G | 3135 | Antigen processing cross presentation | 2.54 |
| CTSS | cathepsin S | 1520 | Antigen processing cross presentation | 1.53 |
| RPS27A | ribosomal protein S27a | 6233 | Antigen processing cross presentation | 1.63 |
| ITGAV | integrin subunit alpha V | 3685 | Antigen processing cross presentation | 2.68 |
| PSMF1 | proteasome inhibitor subunit 1 | 9491 | Antigen processing cross presentation | 1.15 |
| CALR | calreticulin | 811 | Antigen processing cross presentation | 4.30 |
| PSMD3 | proteasome 26S subunit, non-ATPase 3 | 5709 | Antigen processing cross presentation | 1.20 |
| MRC2 | mannose receptor C type 2 | 9902 | Antigen processing cross presentation | 1.34 |
| CYBB | cytochrome b-245 beta chain | 1536 | Antigen processing cross presentation | 1.44 |
| PSMB6 | proteasome 20S subunit beta 6 | 5694 | Antigen processing cross presentation | 1.13 |
| PSMD13 | proteasome 26S subunit, non-ATPase 13 | 5719 | Antigen processing cross presentation | 1.09 |
| SEC61G | SEC61 translocon subunit gamma | 23480 | Antigen processing cross presentation | 3.06 |
| BTK | Bruton tyrosine kinase | 695 | Antigen processing cross presentation | 1.82 |
| PSMB4 | proteasome 20S subunit beta 4 | 5692 | Antigen processing cross presentation | 0.94 |
| PSMB5 | proteasome 20S subunit beta 5 | 5693 | Antigen processing cross presentation | 1.64 |
| PSMD12 | proteasome 26S subunit, non-ATPase 12 | 5718 | Antigen processing cross presentation | 0.84 |
| PSMB10 | proteasome 20S subunit beta 10 | 5699 | Antigen processing cross presentation | 0.52 |
| TAPBP | TAP binding protein | 6892 | Antigen processing cross presentation | 2.21 |
| VAMP3 | vesicle associated membrane protein 3 | 9341 | Antigen processing cross presentation | 1.53 |
| PSMD10 | proteasome 26S subunit, non-ATPase 10 | 5716 | Antigen processing cross presentation | 1.25 |
| IKBKB | inhibitor of nuclear factor kappa B kinase subunit beta | 3551 | Antigen processing cross presentation | 0.77 |
| UBC | ubiquitin C | 7316 | Antigen processing cross presentation | 2.95 |
| TAP1 | transporter 1, ATP binding cassette subfamily B member | 6890 | Peptide presentation CMH I | 37.14 |
| TAP2 | transporter 2, ATP binding cassette subfamily B member | 6891 | Peptide presentation CMH I | 13.23 |
| HLA-H | major histocompatibility complex, class I, H | 3136 | Peptide presentation CMH I | 11.59 |
| PSMB8 | proteasome 20S subunit beta 8 | 5696 | Peptide presentation CMH I | 11.73 |
| HLA-C | major histocompatibility complex, class I, C | 3107 | Peptide presentation CMH I | 38.66 |
| HLA-B | major histocompatibility complex, class I, B | 3106 | Peptide presentation CMH I | 35.12 |
| PSMA4 | proteasome 20S subunit alpha 4 | 5685 | Peptide presentation CMH I | 6.45 |
| FCER1G | Fc fragment of IgE receptor Ig | 2207 | Peptide presentation CMH I | 6.41 |
| PSME1 | proteasome activator subunit 1 | 5720 | Peptide presentation CMH I | 13.94 |
| SEC24A | SEC24 homolog A, COPII coat complex component | 10802 | Peptide presentation CMH I | 7.64 |
| PSMA3 | proteasome 20S subunit alpha 3 | 5684 | Peptide presentation CMH I | 5.91 |
| PSMB9 | proteasome 20S subunit beta 9 | 5698 | Peptide presentation CMH I | 8.47 |
| HLA-A | major histocompatibility complex, class I, A | 3105 | Peptide presentation CMH I | 17.63 |
| B2M | beta-2-microglobulin | 567 | Peptide presentation CMH I | 25.24 |
| SEC24D | SEC24 homolog D, COPII coat complex component | 9871 | Peptide presentation CMH I | 3.11 |
| CHUK | component of inhibitor of nuclear factor kappa B kinase complex | 1147 | Peptide presentation CMH I | 2.96 |
| PSME2 | proteasome activator subunit 2 | 5721 | Peptide presentation CMH I | 4.87 |
| PDIA3 | protein disulfide isomerase family A member 3 | 2923 | Peptide presentation CMH I | 4.46 |
| SEC22B | SEC22 homolog B, vesicle trafficking protein | 9554 | Peptide presentation CMH I | 2.74 |
| PSMA1 | proteasome 20S subunit alpha 1 | 5682 | Peptide presentation CMH I | 4.36 |
| PSMD1 | proteasome 26S subunit, non-ATPase 1 | 5707 | Peptide presentation CMH I | 3.01 |
| HLA-F | major histocompatibility complex, class I, F | 3134 | Peptide presentation CMH I | 5.08 |
| PSMA2 | proteasome 20S subunit alpha 2 | 5683 | Peptide presentation CMH I | 4.33 |
| PSMD7 | proteasome 26S subunit, non-ATPase 7 | 5713 | Peptide presentation CMH I | 2.38 |
| PSMB3 | proteasome 20S subunit beta 3 | 5691 | Peptide presentation CMH I | 2.46 |
| FCGR1A | Fc fragment of IgG receptor Ia | 2209 | Peptide presentation CMH I | 5.51 |
| PSMB1 | proteasome 20S subunit beta 1 | 5689 | Peptide presentation CMH I | 2.19 |
| SEC24B | SEC24 homolog B, COPII coat complex component | 10427 | Peptide presentation CMH I | 2.14 |
| PSMD14 | proteasome 26S subunit, non-ATPase 14 | 10213 | Peptide presentation CMH I | 2.33 |
| PSMC6 | proteasome 26S subunit, ATPase 6 | 5706 | Peptide presentation CMH I | 2.16 |
| PSMD8 | proteasome 26S subunit, non-ATPase 8 | 5714 | Peptide presentation CMH I | 2.46 |
| PSMC3 | proteasome 26S subunit, ATPase 3 | 5702 | Peptide presentation CMH I | 5.25 |
| HLA-E | major histocompatibility complex, class I, E | 3133 | Peptide presentation CMH I | 7.02 |
| PSMA5 | proteasome 20S subunit alpha 5 | 5686 | Peptide presentation CMH I | 2.49 |
| PSMC1 | proteasome 26S subunit, ATPase 1 | 5700 | Peptide presentation CMH I | 2.63 |
| IKBKG | inhibitor of nuclear factor kappa B kinase regulatory subunit gamma | 8517 | Peptide presentation CMH I | 1.45 |
| PSMD2 | proteasome 26S subunit ubiquitin receptor, non-ATPase 2 | 5708 | Peptide presentation CMH I | 1.46 |
| ITGB5 | integrin subunit beta 5 | 3693 | Peptide presentation CMH I | 1.31 |
| SEC24C | SEC24 homolog C, COPII coat complex component | 9632 | Peptide presentation CMH I | 0.79 |
| HLA-G | major histocompatibility complex, class I, G | 3135 | Peptide presentation CMH I | 2.54 |
| ITGAV | integrin subunit alpha V | 3685 | Peptide presentation CMH I | 2.68 |
| PSMF1 | proteasome inhibitor subunit 1 | 9491 | Peptide presentation CMH I | 1.15 |
| SEC23A | SEC23 homolog A, COPII coat complex component | 10484 | Peptide presentation CMH I | 2.49 |
| CALR | calreticulin | 811 | Peptide presentation CMH I | 4.30 |
| PSMD3 | proteasome 26S subunit, non-ATPase 3 | 5709 | Peptide presentation CMH I | 1.20 |
| CYBB | cytochrome b-245 beta chain | 1536 | Peptide presentation CMH I | 1.44 |
| IDE | insulin degrading enzyme | 3416 | Peptide presentation CMH I | 1.16 |
| PSMB6 | proteasome 20S subunit beta 6 | 5694 | Peptide presentation CMH I | 1.13 |
| PSMD13 | proteasome 26S subunit, non-ATPase 13 | 5719 | Peptide presentation CMH I | 1.09 |
| HFE | homeostatic iron regulator | 3077 | Peptide presentation CMH I | 1.15 |
| PSMB4 | proteasome 20S subunit beta 4 | 5692 | Peptide presentation CMH I | 0.94 |
| PSMB5 | proteasome 20S subunit beta 5 | 5693 | Peptide presentation CMH I | 1.64 |
| PSMD12 | proteasome 26S subunit, non-ATPase 12 | 5718 | Peptide presentation CMH I | 0.84 |
| PSMB10 | proteasome 20S subunit beta 10 | 5699 | Peptide presentation CMH I | 0.52 |
| TAPBP | TAP binding protein | 6892 | Peptide presentation CMH I | 2.21 |
| SEC31A | SEC31 homolog A, COPII coat complex component | 22872 | Peptide presentation CMH I | 2.23 |
| VAMP3 | vesicle associated membrane protein 3 | 9341 | Peptide presentation CMH I | 1.53 |
| PSMD10 | proteasome 26S subunit, non-ATPase 10 | 5716 | Peptide presentation CMH I | 1.25 |
| IKBKB | inhibitor of nuclear factor kappa B kinase subunit beta | 3551 | Peptide presentation CMH I | 0.77 |
| TAP1 | transporter 1, ATP binding cassette subfamily B member | 6890 | Exogenous Antigen presentation CMH I | 37.14 |
| TAP2 | transporter 2, ATP binding cassette subfamily B member | 6891 | Exogenous Antigen presentation CMH I | 13.23 |
| HLA-H | major histocompatibility complex, class I, H | 3136 | Exogenous Antigen presentation CMH I | 11.59 |
| PSMB8 | proteasome 20S subunit beta 8 | 5696 | Exogenous Antigen presentation CMH I | 11.73 |
| HLA-C | major histocompatibility complex, class I, C | 3107 | Exogenous Antigen presentation CMH I | 38.66 |
| HLA-B | major histocompatibility complex, class I, B | 3106 | Exogenous Antigen presentation CMH I | 35.12 |
| PSMA4 | proteasome 20S subunit alpha 4 | 5685 | Exogenous Antigen presentation CMH I | 6.45 |
| FCER1G | Fc fragment of IgE receptor Ig | 2207 | Exogenous Antigen presentation CMH I | 6.41 |
| PSME1 | proteasome activator subunit 1 | 5720 | Exogenous Antigen presentation CMH I | 13.94 |
| PSMA3 | proteasome 20S subunit alpha 3 | 5684 | Exogenous Antigen presentation CMH I | 5.91 |
| PSMB9 | proteasome 20S subunit beta 9 | 5698 | Exogenous Antigen presentation CMH I | 8.47 |
| HLA-A | major histocompatibility complex, class I, A | 3105 | Exogenous Antigen presentation CMH I | 17.63 |
| B2M | beta-2-microglobulin | 567 | Exogenous Antigen presentation CMH I | 25.24 |
| CHUK | component of inhibitor of nuclear factor kappa B kinase complex | 1147 | Exogenous Antigen presentation CMH I | 2.96 |
| PSME2 | proteasome activator subunit 2 | 5721 | Exogenous Antigen presentation CMH I | 4.87 |
| PDIA3 | protein disulfide isomerase family A member 3 | 2923 | Exogenous Antigen presentation CMH I | 4.46 |
| SEC22B | SEC22 homolog B, vesicle trafficking protein | 9554 | Exogenous Antigen presentation CMH I | 2.74 |
| PSMA1 | proteasome 20S subunit alpha 1 | 5682 | Exogenous Antigen presentation CMH I | 4.36 |
| PSMD1 | proteasome 26S subunit, non-ATPase 1 | 5707 | Exogenous Antigen presentation CMH I | 3.01 |
| HLA-F | major histocompatibility complex, class I, F | 3134 | Exogenous Antigen presentation CMH I | 5.08 |
| PSMA2 | proteasome 20S subunit alpha 2 | 5683 | Exogenous Antigen presentation CMH I | 4.33 |
| PSMD7 | proteasome 26S subunit, non-ATPase 7 | 5713 | Exogenous Antigen presentation CMH I | 2.38 |
| PSMB3 | proteasome 20S subunit beta 3 | 5691 | Exogenous Antigen presentation CMH I | 2.46 |
| FCGR1A | Fc fragment of IgG receptor Ia | 2209 | Exogenous Antigen presentation CMH I | 5.51 |
| PSMB1 | proteasome 20S subunit beta 1 | 5689 | Exogenous Antigen presentation CMH I | 2.19 |
| PSMD14 | proteasome 26S subunit, non-ATPase 14 | 10213 | Exogenous Antigen presentation CMH I | 2.33 |
| PSMC6 | proteasome 26S subunit, ATPase 6 | 5706 | Exogenous Antigen presentation CMH I | 2.16 |
| PSMD8 | proteasome 26S subunit, non-ATPase 8 | 5714 | Exogenous Antigen presentation CMH I | 2.46 |
| PSMC3 | proteasome 26S subunit, ATPase 3 | 5702 | Exogenous Antigen presentation CMH I | 5.25 |
| HLA-E | major histocompatibility complex, class I, E | 3133 | Exogenous Antigen presentation CMH I | 7.02 |
| PSMA5 | proteasome 20S subunit alpha 5 | 5686 | Exogenous Antigen presentation CMH I | 2.49 |
| PSMC1 | proteasome 26S subunit, ATPase 1 | 5700 | Exogenous Antigen presentation CMH I | 2.63 |
| IKBKG | inhibitor of nuclear factor kappa B kinase regulatory subunit gamma | 8517 | Exogenous Antigen presentation CMH I | 1.45 |
| PSMD2 | proteasome 26S subunit ubiquitin receptor, non-ATPase 2 | 5708 | Exogenous Antigen presentation CMH I | 1.46 |
| ITGB5 | integrin subunit beta 5 | 3693 | Exogenous Antigen presentation CMH I | 1.31 |
| HLA-G | major histocompatibility complex, class I, G | 3135 | Exogenous Antigen presentation CMH I | 2.54 |
| ITGAV | integrin subunit alpha V | 3685 | Exogenous Antigen presentation CMH I | 2.68 |
| PSMF1 | proteasome inhibitor subunit 1 | 9491 | Exogenous Antigen presentation CMH I | 1.15 |
| CALR | calreticulin | 811 | Exogenous Antigen presentation CMH I | 4.30 |
| PSMD3 | proteasome 26S subunit, non-ATPase 3 | 5709 | Exogenous Antigen presentation CMH I | 1.20 |
| CYBB | cytochrome b-245 beta chain | 1536 | Exogenous Antigen presentation CMH I | 1.44 |
| PSMB6 | proteasome 20S subunit beta 6 | 5694 | Exogenous Antigen presentation CMH I | 1.13 |
| PSMD13 | proteasome 26S subunit, non-ATPase 13 | 5719 | Exogenous Antigen presentation CMH I | 1.09 |
| PSMB4 | proteasome 20S subunit beta 4 | 5692 | Exogenous Antigen presentation CMH I | 0.94 |
| PSMB5 | proteasome 20S subunit beta 5 | 5693 | Exogenous Antigen presentation CMH I | 1.64 |
| PSMD12 | proteasome 26S subunit, non-ATPase 12 | 5718 | Exogenous Antigen presentation CMH I | 0.84 |
| PSMB10 | proteasome 20S subunit beta 10 | 5699 | Exogenous Antigen presentation CMH I | 0.52 |
| TAPBP | TAP binding protein | 6892 | Exogenous Antigen presentation CMH I | 2.21 |
| VAMP3 | vesicle associated membrane protein 3 | 9341 | Exogenous Antigen presentation CMH I | 1.53 |
| PSMD10 | proteasome 26S subunit, non-ATPase 10 | 5716 | Exogenous Antigen presentation CMH I | 1.25 |
| IKBKB | inhibitor of nuclear factor kappa B kinase subunit beta | 3551 | Exogenous Antigen presentation CMH I | 0.77 |

**Supplemental Table 5: Genes identified as significant in the PSMB8-defined cell trajectory of CD14+/CD68+ bronchoalveolar cells from COVID-19 patients.** Columns describe respective gene symbol, their p-values and their corrected q-values found on the trajectory of single cell transcriptome for M1 pro-inflammatory macrophage found in bronchoalveolar fluid lavages of the patients

| **gene names** | **p-values** | **Corrected q-values** |
| --- | --- | --- |
| SPP1 | 0 | 0 |
| FABP4 | 0 | 0 |
| NUPR1 | 0 | 0 |
| RBP4 | 2.26308798736478e-213 | 1.32407620420745e-209 |
| HLA-DQA2 | 1.66285931286392e-156 | 7.78317929979088e-153 |
| CCL3L1 | 1.22726445678764e-151 | 4.78694501370018e-148 |
| CCL4L2 | 1.0585860307647e-145 | 3.53915555399806e-142 |
| APOC1 | 2.24199235720478e-131 | 6.55866839195795e-128 |
| CES1 | 4.82738229154008e-131 | 1.25528030854347e-127 |
| CCL2 | 1.03424415187508e-123 | 2.42044158863325e-120 |
| GCHFR | 4.6868362986898e-116 | 9.97145726347612e-113 |
| CXCL8 | 1.97772780703782e-110 | 3.85706365567551e-107 |
| S100A8 | 7.42977265818246e-102 | 1.33753053476495e-98 |
| APOE | 4.37593963193944e-100 | 7.3150082290199e-97 |
| CCL4 | 4.81480348650121e-100 | 7.51205639963919e-97 |
| TIMP1 | 2.39430521150323e-97 | 3.50212030405063e-94 |
| FN1 | 2.8824239372755e-89 | 3.96808043553285e-86 |
| CYP27A1 | 8.72175334884601e-86 | 1.1339732979058e-82 |
| CCL7 | 1.03180321942631e-85 | 1.27091003917021e-82 |
| NEAT1 | 6.76948533110605e-81 | 7.92131326019374e-78 |
| MT1M | 2.38793667700512e-79 | 2.6611848596167e-76 |
| MT-ND4 | 1.40443821721388e-72 | 1.49400307261165e-69 |
| S100A9 | 1.16691293853262e-68 | 1.18735928262951e-65 |
| SLPI | 5.82273950085631e-67 | 5.67789885577251e-64 |
| IDO1 | 7.20041038517118e-67 | 6.74044816976645e-64 |
| MT2A | 1.43740200117794e-66 | 1.29382765513721e-63 |
| FOS | 1.30264492241792e-64 | 1.12910367108691e-61 |
| CXCL2 | 1.66003135146808e-64 | 1.38748977565741e-61 |
| ALDH2 | 4.47142763252919e-61 | 3.60844209945106e-58 |
| RMDN3 | 9.37535015018663e-61 | 7.31371065216059e-58 |
| TNFSF13 | 3.89583667054979e-59 | 2.94110534196376e-56 |
| MARCO | 2.44494727139793e-58 | 1.78809690601643e-55 |
| DUSP1 | 1.97631222400509e-57 | 1.40156469631488e-54 |
| S100A12 | 3.10673568254752e-57 | 2.13843926996058e-54 |
| GPNMB | 6.11422793265717e-57 | 4.08832218022788e-54 |
| PLIN2 | 8.80274672542868e-56 | 5.72251893375576e-53 |
| RGCC | 2.66099896980529e-55 | 1.63882523395666e-52 |
| IFI27 | 2.61319866966109e-55 | 1.63882523395666e-52 |
| MDK | 2.11032930743642e-53 | 1.2663599174855e-50 |
| SCGB3A1 | 2.77108185984469e-53 | 1.62129071914863e-50 |
| PLA2G16 | 6.44218797954193e-52 | 3.67723232402975e-49 |
| FCN1 | 1.13594354103007e-50 | 6.32963968826828e-48 |
| GRN | 5.43355212150177e-50 | 2.95724233254665e-47 |
| CXCL9 | 1.6597646402979e-49 | 8.82806179020268e-47 |
| MT-CO2 | 2.85405753070339e-49 | 1.48430018646781e-46 |
| HMOX1 | 6.06350536445289e-48 | 3.08487426183241e-45 |
| CXCL3 | 7.03123415708147e-48 | 3.50110580804633e-45 |
| IL1R2 | 1.60267572377733e-46 | 7.81404582574183e-44 |
| CCL3 | 3.41643947374453e-46 | 1.63173332661313e-43 |
| S100A4 | 4.11457908270027e-46 | 1.92586988544869e-43 |
| WARS | 6.99948768473733e-46 | 3.21194137815505e-43 |
| PLD3 | 2.57539864865012e-45 | 1.15907797258382e-42 |
| SDS | 4.56496726002435e-45 | 2.01573450540283e-42 |
| MT-ND3 | 1.27620289868089e-44 | 5.5309215625609e-42 |
| IFIT2 | 3.41691396876516e-44 | 1.45392795656384e-41 |
| PARAL1 | 2.68452653198291e-43 | 1.12189240049993e-40 |
| TUBB | 1.00721417804957e-42 | 4.1354093699814e-40 |
| CAPG | 1.46481924994677e-42 | 5.91054567353522e-40 |
| LGMN | 1.66525546648528e-42 | 6.60541926816186e-40 |
| IER3 | 2.81800745691047e-42 | 1.09916380856793e-39 |
| ZFP36 | 2.52185717350763e-41 | 9.6752497428851e-39 |
| TAOK1 | 4.41059117179039e-41 | 1.6648558902163e-38 |
| CXCL11 | 5.00801108306711e-41 | 1.86035687900031e-38 |
| KLHDC8B | 6.07416419144658e-41 | 2.22115100894413e-38 |
| IL1RN | 2.32654198242571e-40 | 8.37662492533984e-38 |
| CD68 | 1.42692689305717e-39 | 5.05975304215408e-37 |
| FOSB | 1.54388210197461e-39 | 5.39275713918088e-37 |
| NFKBIA | 5.29180455133891e-39 | 1.82123679286742e-36 |
| CLU | 1.5547810888855e-38 | 5.27341185843295e-36 |
| CALHM6 | 6.43867667723145e-38 | 2.15263357538925e-35 |
| HAMP | 1.05418484740017e-36 | 3.47480112446565e-34 |
| RNASE1 | 1.34601269335208e-36 | 4.37510209201648e-34 |
| EMP1 | 4.64334830885307e-36 | 1.48860658180943e-33 |
| TUBA1B | 5.38102997439974e-36 | 1.70178708771456e-33 |
| ACOT2 | 2.39553363187225e-35 | 7.47502314489416e-33 |
| MT-CYB | 2.60282245081023e-35 | 8.01498076530419e-33 |
| LAMP1 | 3.9427066894605e-35 | 1.19832681368109e-32 |
| SGK1 | 1.40124829450238e-34 | 4.20428382515887e-32 |
| C1QB | 3.77813701516132e-34 | 1.11923722235216e-31 |
| SERPING1 | 5.42600524311266e-34 | 1.58731000880707e-31 |
| OAS1 | 9.35488280439775e-34 | 2.70286817618914e-31 |
| AGRP | 1.58005283213246e-33 | 4.50950932078e-31 |
| MT1G | 1.82698498945e-33 | 5.15143731422873e-31 |
| SCGB1A1 | 4.20176265906296e-33 | 1.17064108940536e-30 |
| VAMP5 | 8.44732950698542e-33 | 2.32579826414094e-30 |
| WFDC2 | 1.94905190327198e-32 | 5.30391415026444e-30 |
| SRGN | 3.20773847491997e-32 | 8.62881649753472e-30 |
| PPIC | 9.52931235408785e-32 | 2.53425564798543e-29 |
| LGALS3BP | 2.1784261614985e-31 | 5.72828173680329e-29 |
| COMT | 3.77573596765338e-31 | 9.81817209455467e-29 |
| MME | 6.58230959485251e-31 | 1.69281089503663e-28 |
| CCL8 | 7.46365009042676e-31 | 1.89860655506802e-28 |
| HLA-C | 8.05557213468143e-31 | 2.02714574911774e-28 |
| C1QC | 8.82853654333522e-31 | 2.17488674445973e-28 |
| JUN | 8.82591558223709e-31 | 2.17488674445973e-28 |
| KLF4 | 9.1287089883268e-31 | 2.22540808806054e-28 |
| SCD | 3.50921755290422e-30 | 8.46662045264098e-28 |
| S100A10 | 3.64486975733142e-30 | 8.7041721357987e-28 |
| TREM2 | 6.94793979515107e-30 | 1.64245085884768e-27 |
| MCEMP1 | 1.05990336219744e-29 | 2.48049183855067e-27 |

**Supplemental Table 6: Prevalence of the 69 most-frequent HLA-A,B,C alleles in 20 ethnicities with a worldwide distribution (USA NMDP bone marrow registry).** MHC class I allele frequencies collected for 20 regions in the world and for 69 more prevalent alleles

| **USA_NMDP** | **world region** | **African** | **African American pop2** | **Alaska Native or Aleut** | **American Indian South or Central America** | **Caribean Black** | **Caribean Hispanic** | **Caribean Indian** | **Chinese** | **European Caucasian** | **Filipino** | **Hawaiian or other Pacific Islander** | **Hispanic South or Central American** | **Japanese** | **Korean** | **Mexican or Chicano** | **Middle Eastern or North Coast of Africa** | **North American Amerindian** | **South AsianIndian** | **South east Asian** | **Vietnamese** |
| --- | --- | --- | --- | --- | --- | --- | --- | --- | --- | --- | --- | --- | --- | --- | --- | --- | --- | --- | --- | --- | --- |
| **effectif** | **2790874** | 28557 | 416581 | 1376 | 5926 | 33328 | 115374 | 14339 | 99672 | 1242890 | 50614 | 11499 | 146714 | 24582 | 77584 | 261235 | 7089 | 35791 | 185391 | 27978 | 4354 |
| HLA-A*01:01 | occident | 0.0506 | 0.0467 | 0.105 | 0.119 | 0.0449 | 0.0666 | 0.063 | 0.0145 | 0.1646 | 0.0125 | 0.0447 | 0.0726 | 0.01 | 0.0208 | 0.0735 | 0.135 | 0.1202 | 0.1545 | 0.1147 | 0.0332 |
| HLA-A*02:01 | occident | 0.1146 | 0.1235 | 0.192 | 0.265 | 0.1107 | 0.1688 | 0.145 | 0.0946 | 0.2755 | 0.0671 | 0.1198 | 0.2095 | 0.148 | 0.1857 | 0.223 | 0.1973 | 0.2776 | 0.0492 | 0.0578 | 0.0349 |
| HLA-A*02:06 | asia | 0.0003 | 0.0007 | 0.103 | 0.02 | 0.0003 | 0.0024 | 0.004 | 0.0349 | 0.0018 | 0.0278 | 0.0847 | 0.0198 | 0.0748 | 0.0747 | 0.0504 | 0.002 | 0.0275 | 0.0175 | 0.0189 | 0.0406 |
| HLA-A*03:01 | occident | 0.0783 | 0.0839 | 0.077 | 0.106 | 0.0783 | 0.0863 | 0.081 | 0.014 | 0.1399 | 0.0131 | 0.0323 | 0.0738 | 0.009 | 0.0191 | 0.0815 | 0.101 | 0.1044 | 0.0636 | 0.0499 | 0.0097 |
| HLA-A*11:01 | asia | 0.0112 | 0.0142 | 0.036 | 0.051 | 0.0156 | 0.0412 | 0.044 | 0.2752 | 0.0609 | 0.1792 | 0.1357 | 0.0456 | 0.0874 | 0.1021 | 0.0484 | 0.0641 | 0.0488 | 0.1396 | 0.1753 | 0.2466 |
| HLA-A*23:01 | africa | 0.1047 | 0.1099 | 0.012 | 0.024 | 0.1068 | 0.054 | 0.058 | 0.0021 | 0.0197 | 0.0031 | 0.0063 | 0.0368 | 0.0011 | 0.0003 | 0.0264 | 0.0297 | 0.0181 | 0.0066 | 0.0061 | 0.0007 |
| HLA-A*24:02 | asia | 0.022 | 0.0245 | 0.229 | 0.112 | 0.0251 | 0.0938 | 0.095 | 0.1519 | 0.0846 | 0.2399 | 0.2393 | 0.1316 | 0.353 | 0.2211 | 0.1304 | 0.1174 | 0.1292 | 0.1362 | 0.138 | 0.1109 |
| HLA-A*25:01 | occident | 0.0023 | 0.0034 | 0.007 | 0.014 | 0.0028 | 0.0073 | 0.005 | 0.0002 | 0.021 | 0.0012 | 0.002 | 0.0101 | 0.0007 | 0.0001 | 0.0113 | 0.0097 | 0.0148 | 0.0001 | 0.0003 | 0.0002 |
| HLA-A*26:01 | world | 0.0124 | 0.0146 | 0.021 | 0.024 | 0.0123 | 0.03 | 0.027 | 0.0237 | 0.0309 | 0.0152 | 0.0438 | 0.0295 | 0.0798 | 0.0375 | 0.0259 | 0.0442 | 0.0199 | 0.0418 | 0.0387 | 0.0228 |
| HLA-A*29:02 | occident | 0.0303 | 0.0318 | 0.02 | 0.031 | 0.0293 | 0.0607 | 0.056 | 0.001 | 0.0353 | 0.0029 | 0.0107 | 0.0441 | 0.0018 | 0.0007 | 0.039 | 0.0193 | 0.0274 | 0.0013 | 0.0022 | 0.005 |
| HLA-A*30:01 | world | 0.0685 | 0.0685 | 0.009 | 0.014 | 0.072 | 0.0294 | 0.03 | 0.0274 | 0.013 | 0.0045 | 0.0029 | 0.0199 | 0.0019 | 0.0317 | 0.0162 | 0.0283 | 0.0109 | 0.0173 | 0.0169 | 0.0139 |
| HLA-A*30:02 | africa | 0.0654 | 0.0667 | 0.006 | 0.016 | 0.0694 | 0.0317 | 0.031 | 0.0003 | 0.009 | 0.0014 | 0.0047 | 0.0273 | 0.0006 | 0.0001 | 0.023 | 0.0133 | 0.0116 | 0.0024 | 0.0023 | 0.0002 |
| HLA-A*31:01 | world | 0.0079 | 0.01 | 0.052 | 0.054 | 0.0094 | 0.0289 | 0.029 | 0.0242 | 0.027 | 0.0059 | 0.0109 | 0.0439 | 0.0849 | 0.0535 | 0.0531 | 0.0186 | 0.0737 | 0.0331 | 0.0259 | 0.0137 |
| HLA-A*32:01 | occident | 0.0154 | 0.015 | 0.025 | 0.028 | 0.0133 | 0.0337 | 0.029 | 0.0062 | 0.0355 | 0.0028 | 0.011 | 0.0261 | 0.0017 | 0.0061 | 0.0247 | 0.0493 | 0.0254 | 0.0386 | 0.0299 | 0.0017 |
| HLA-A*33:03 | asia | 0.0523 | 0.0518 | 0.004 | 0.008 | 0.0567 | 0.0149 | 0.029 | 0.1011 | 0.0032 | 0.0511 | 0.0238 | 0.0082 | 0.0648 | 0.1529 | 0.0049 | 0.0109 | 0.0039 | 0.0992 | 0.0948 | 0.1188 |
| HLA-A*68:01 | world | 0.0378 | 0.0395 | 0.064 | 0.042 | 0.0369 | 0.0385 | 0.043 | 0.004 | 0.0319 | 0.0032 | 0.0136 | 0.048 | 0.0017 | 0.0024 | 0.0518 | 0.03 | 0.0381 | 0.0676 | 0.0499 | 0.0057 |
| HLA-A*68:02 | africa | 0.0645 | 0.0603 | 0.006 | 0.012 | 0.0636 | 0.044 | 0.049 | 0.0001 | 0.0084 | 0.0009 | 0.0045 | 0.0248 | 0.0003 | 0.000071 | 0.0185 | 0.0112 | 0.0079 | 0.0003 | 0.0008 | 0.0004 |
| HLA-A*74:01 | africa | 0.0567 | 0.0546 | 0.001 | 0.003 | 0.0558 | 0.0136 | 0.018 | 0.0014 | 0.0004 | 0.0005 | 0.0014 | 0.0073 | 0.0001 | 0.000045 | 0.0044 | 0.0011 | 0.0023 | 0.0015 | 0.0024 | 0.0028 |
| HLA-B*07:02 | world | 0.0689 | 0.0729 | 0.078 | 0.096 | 0.0729 | 0.0568 | 0.051 | 0.0079 | 0.1306 | 0.0099 | 0.0287 | 0.0584 | 0.0588 | 0.0344 | 0.0574 | 0.0565 | 0.1005 | 0.044 | 0.0334 | 0.017 |
| HLA-B*08:01 | occident | 0.032 | 0.0376 | 0.069 | 0.08 | 0.0286 | 0.0414 | 0.035 | 0.0046 | 0.1144 | 0.0062 | 0.0213 | 0.039 | 0.0051 | 0.0048 | 0.0422 | 0.0536 | 0.0822 | 0.0366 | 0.0321 | 0.0048 |
| HLA-B*13:01 | asia | 0.0001 | 0.000097 | 0.001 | NA | 0.0006 | 0.0001 | 0.001 | 0.0635 | 0.0000961 | 0.028 | 0.0199 | 0.0002 | 0.0111 | 0.0216 | 0.0001 | 0.0005 | 0.0002 | 0.0136 | 0.028 | 0.0394 |
| HLA-B*13:02 | world | 0.0123 | 0.0078 | 0.013 | 0.017 | 0.008 | 0.0088 | 0.007 | 0.0291 | 0.0239 | 0.0048 | 0.0049 | 0.0128 | 0.0037 | 0.0321 | 0.0138 | 0.0319 | 0.0164 | 0.0184 | 0.0181 | 0.0077 |
| HLA-B*14:02 | occident | 0.0219 | 0.0216 | 0.014 | 0.026 | 0.0212 | 0.039 | 0.032 | 0.001 | 0.0286 | 0.0029 | 0.0092 | 0.0441 | 0.0012 | 0.0002 | 0.0428 | 0.0361 | 0.0204 | 0.0017 | 0.0026 | 0.0004 |
| HLA-B*15:01 | world | 0.0066 | 0.0108 | 0.085 | 0.051 | 0.009 | 0.0234 | 0.021 | 0.03 | 0.0606 | 0.0132 | 0.0201 | 0.0271 | 0.0769 | 0.0839 | 0.0306 | 0.0233 | 0.0616 | 0.0161 | 0.0161 | 0.0108 |
| HLA-B*15:02 | asia | 0.0004 | 0.0003 | 0.002 | 0.001 | 0.0009 | 0.0002 | 0.002 | 0.0647 | 0.000041 | 0.0421 | 0.0081 | 0.0003 | 0.0006 | 0.0034 | 0.0003 | 0.0004 | 0.0002 | 0.0256 | 0.0403 | 0.1383 |
| HLA-B*15:25 | asia | 0.0001 | 0.0001 | NA | NA | 0.0002 | 0.0004 | 0.001 | 0.0074 | 0.0000072 | 0.003 | 0.0011 | 0.000092 | 0.0002 | 0.0004 | 0.000048 | 0.000063 | 0.00006 | 0.0051 | 0.0078 | 0.0505 |
| HLA-B*18:01 | occident | 0.0319 | 0.032 | 0.026 | 0.039 | 0.0315 | 0.0427 | 0.035 | 0.0026 | 0.0443 | 0.0246 | 0.0168 | 0.0411 | 0.0021 | 0.0006 | 0.0409 | 0.0724 | 0.0323 | 0.0252 | 0.0294 | 0.0108 |
| HLA-B*27:02 | occident | 0.0005 | 0.0004 | 0.001 | 0.002 | 0.0004 | 0.0035 | 0.002 | 0.00007 | 0.0039 | 0.0001 | 0.0004 | 0.0019 | 0.0002 | 0.000058 | 0.0019 | 0.0059 | 0.0028 | 0.0008 | 0.0009 | 0.000002 |
| HLA-B*27:05 | occident | 0.0059 | 0.0084 | 0.089 | 0.04 | 0.0067 | 0.0172 | 0.016 | 0.0035 | 0.0373 | 0.0032 | 0.0076 | 0.016 | 0.0023 | 0.0249 | 0.0222 | 0.0148 | 0.0545 | 0.0083 | 0.0071 | 0.0024 |
| HLA-B*35:01 | occident | 0.0668 | 0.0689 | 0.087 | 0.081 | 0.0734 | 0.0479 | 0.044 | 0.0223 | 0.056 | 0.0225 | 0.0203 | 0.0713 | 0.0869 | 0.0599 | 0.0798 | 0.0649 | 0.0979 | 0.0622 | 0.0476 | 0.0101 |
| HLA-B*35:03 | world | 0.0016 | 0.002 | 0.011 | 0.011 | 0.0043 | 0.0238 | 0.03 | 0.0079 | 0.0162 | 0.0044 | 0.0077 | 0.0147 | 0.0005 | 0.0046 | 0.013 | 0.031 | 0.0095 | 0.072 | 0.0512 | 0.0057 |
| HLA-B*37:01 | world | 0.0062 | 0.0055 | 0.01 | 0.012 | 0.0049 | 0.0088 | 0.01 | 0.006 | 0.0144 | 0.0026 | 0.0045 | 0.0075 | 0.0048 | 0.0158 | 0.0073 | 0.0111 | 0.0108 | 0.0341 | 0.0251 | 0.0078 |
| HLA-B*38:01 | occident | 0.0026 | 0.0022 | 0.007 | 0.013 | 0.0026 | 0.0182 | 0.016 | 0.0041 | 0.0209 | 0.0017 | 0.0034 | 0.0199 | 0.001 | 0.0012 | 0.0183 | 0.0369 | 0.0102 | 0.0028 | 0.0043 | 0.0019 |
| HLA-B*39:01 | world | 0.0018 | 0.0032 | 0.015 | 0.021 | 0.0018 | 0.0074 | 0.01 | 0.0193 | 0.0112 | 0.0107 | 0.0393 | 0.0073 | 0.0303 | 0.0146 | 0.0082 | 0.0098 | 0.0339 | 0.0055 | 0.0074 | 0.0087 |
| HLA-B*40:01 | world | 0.0087 | 0.0128 | 0.046 | 0.044 | 0.0097 | 0.017 | 0.015 | 0.1538 | 0.0528 | 0.0924 | 0.1543 | 0.0144 | 0.0603 | 0.042 | 0.0143 | 0.0206 | 0.0518 | 0.0221 | 0.045 | 0.0533 |
| HLA-B*40:02 | world | 0.0027 | 0.0033 | 0.116 | 0.03 | 0.0035 | 0.0301 | 0.028 | 0.0173 | 0.0126 | 0.0771 | 0.0482 | 0.0478 | 0.0766 | 0.0467 | 0.0573 | 0.0093 | 0.031 | 0.003 | 0.0069 | 0.0076 |
| HLA-B*44:02 | occident | 0.0131 | 0.0208 | 0.057 | 0.072 | 0.0158 | 0.0424 | 0.031 | 0.0038 | 0.0952 | 0.0054 | 0.0173 | 0.0403 | 0.0075 | 0.0162 | 0.0413 | 0.0542 | 0.0744 | 0.0082 | 0.0085 | 0.0015 |
| HLA-B*44:03 | world | 0.0424 | 0.0459 | 0.025 | 0.044 | 0.0577 | 0.0894 | 0.095 | 0.014 | 0.0467 | 0.0155 | 0.028 | 0.0552 | 0.0605 | 0.085 | 0.047 | 0.03 | 0.0378 | 0.074 | 0.0609 | 0.0275 |
| HLA-B*46:01 | asia | 0.00007 | 0.000072 | NA | NA | 0.0006 | 0.0002 | NA | 0.1343 | 0.0000752 | 0.0251 | 0.0104 | 0.0002 | 0.041 | 0.0507 | 0.0002 | 0.0003 | 0.0001 | 0.001 | 0.0341 | 0.1194 |
| HLA-B*48:01 | asia | 0.0004 | 0.0005 | 0.057 | 0.012 | 0.0004 | 0.0021 | 0.002 | 0.0131 | 0.0007 | 0.0266 | 0.0694 | 0.0142 | 0.0271 | 0.0367 | 0.0254 | 0.0009 | 0.0182 | 0.0037 | 0.0044 | 0.006 |
| HLA-B*49:01 | world | 0.038 | 0.0279 | 0.011 | 0.017 | 0.0263 | 0.0264 | 0.022 | 0.0006 | 0.0158 | 0.002 | 0.0049 | 0.0257 | 0.0005 | 0.0003 | 0.0225 | 0.0377 | 0.0145 | 0.0061 | 0.0061 | 0.0002 |
| HLA-B*50:01 | occident | 0.0117 | 0.0089 | 0.008 | 0.009 | 0.0088 | 0.0207 | 0.02 | 0.0027 | 0.0105 | 0.0013 | 0.0033 | 0.015 | 0.0005 | 0.0014 | 0.0133 | 0.0297 | 0.0079 | 0.0172 | 0.0152 | 0.0021 |
| HLA-B*51:01 | world | 0.0241 | 0.0217 | 0.057 | 0.056 | 0.0226 | 0.0638 | 0.062 | 0.0457 | 0.0473 | 0.0376 | 0.0236 | 0.0605 | 0.089 | 0.0918 | 0.0578 | 0.0781 | 0.0678 | 0.0747 | 0.067 | 0.0228 |
| HLA-B*52:01 | world | 0.0172 | 0.0154 | 0.007 | 0.014 | 0.0204 | 0.0213 | 0.032 | 0.0134 | 0.0089 | 0.0057 | 0.0103 | 0.0205 | 0.099 | 0.0252 | 0.026 | 0.0331 | 0.0066 | 0.0745 | 0.0592 | 0.0086 |
| HLA-B*53:01 | africa | 0.1213 | 0.1178 | 0.002 | 0.011 | 0.1281 | 0.0287 | 0.032 | 0.0003 | 0.0034 | 0.0008 | 0.004 | 0.0195 | 0.0003 | 0.0001 | 0.0123 | 0.0121 | 0.0073 | 0.0013 | 0.0014 | 0.0002 |
| HLA-B*55:01 | occident | 0.0028 | 0.004 | 0.011 | 0.014 | 0.004 | 0.0087 | 0.007 | 0.0008 | 0.0186 | 0.0011 | 0.0055 | 0.0091 | 0.001 | 0.0007 | 0.009 | 0.0219 | 0.0143 | 0.0203 | 0.0155 | 0.0008 |
| HLA-B*56:01 | asia | 0.0018 | 0.0023 | 0.005 | 0.004 | 0.0022 | 0.0073 | 0.009 | 0.0061 | 0.0064 | 0.0034 | 0.0261 | 0.0034 | 0.0093 | 0.0042 | 0.0041 | 0.0036 | 0.0044 | 0.0061 | 0.0063 | 0.0068 |
| HLA-B*57:01 | world | 0.0058 | 0.0071 | 0.019 | 0.029 | 0.0067 | 0.0153 | 0.02 | 0.0051 | 0.0365 | 0.0039 | 0.0161 | 0.0152 | 0.0013 | 0.0042 | 0.0127 | 0.0219 | 0.0268 | 0.0676 | 0.0479 | 0.0257 |
| HLA-B*58:01 | asia | 0.0425 | 0.0378 | 0.006 | 0.01 | 0.0405 | 0.0233 | 0.026 | 0.0874 | 0.0073 | 0.0404 | 0.0145 | 0.0142 | 0.0076 | 0.0603 | 0.009 | 0.0173 | 0.007 | 0.042 | 0.0478 | 0.0692 |
| HLA-B*58:02 | africa | 0.0376 | 0.0422 | NA | 0.004 | 0.0316 | 0.007 | 0.009 | 0.000035 | 0.0001 | 0.000079 | 0.0005 | 0.0037 | NA | 0.000006 | 0.0024 | 0.0002 | 0.0018 | 0.000057 | 0.0001 | 0.000069 |
| HLA-C*01:02 | asia | 0.0048 | 0.0078 | 0.053 | 0.046 | 0.0086 | 0.0237 | 0.025 | 0.1915 | 0.0341 | 0.0465 | 0.1291 | 0.0544 | 0.1732 | 0.1677 | 0.0507 | 0.026 | 0.0492 | 0.0351 | 0.069 | 0.1673 |
| HLA-C*02:02 | world | 0.0847 | 0.089 | 0.088 | 0.043 | 0.0807 | 0.0624 | 0.064 | 0.0036 | 0.0435 | 0.0029 | 0.0064 | 0.0367 | 0.0015 | 0.007 | 0.0336 | 0.0348 | 0.0554 | 0.0093 | 0.0081 | 0.0018 |
| HLA-C*02:09 | unknown | NA | NA | NA | NA | NA | NA | NA | NA | NA | NA | NA | NA | NA | NA | NA | NA | NA | NA | NA | NA |
| HLA-C*03:02 | asia | 0.0177 | 0.0185 | 0.005 | 0.006 | 0.02 | 0.0065 | 0.014 | 0.0874 | 0.0022 | 0.0402 | 0.0135 | 0.0049 | 0.0076 | 0.0628 | 0.0037 | 0.008 | 0.0023 | 0.0419 | 0.0473 | 0.0719 |
| HLA-C*03:03 | asia | 0.0091 | 0.0129 | 0.044 | 0.044 | 0.0118 | 0.0292 | 0.025 | 0.0473 | 0.0534 | 0.0273 | 0.024 | 0.0298 | 0.1485 | 0.1195 | 0.0366 | 0.0289 | 0.0417 | 0.0152 | 0.0203 | 0.0397 |
| HLA-C*03:04 | asia | 0.0486 | 0.0565 | 0.206 | 0.082 | 0.0497 | 0.0452 | 0.045 | 0.1165 | 0.0749 | 0.0646 | 0.1173 | 0.0602 | 0.1269 | 0.1013 | 0.0726 | 0.0284 | 0.1016 | 0.0162 | 0.044 | 0.072 |
| HLA-C*04:01 | world | 0.2072 | 0.2037 | 0.111 | 0.134 | 0.2279 | 0.1486 | 0.152 | 0.0434 | 0.1059 | 0.1716 | 0.1558 | 0.1756 | 0.0404 | 0.0556 | 0.171 | 0.1689 | 0.1312 | 0.1364 | 0.1148 | 0.0383 |
| HLA-C*05:01 | occident | 0.0246 | 0.0336 | 0.062 | 0.079 | 0.0285 | 0.0556 | 0.036 | 0.0036 | 0.0939 | 0.0054 | 0.0184 | 0.0579 | 0.0072 | 0.0162 | 0.0575 | 0.0452 | 0.0761 | 0.0084 | 0.0086 | 0.0015 |
| HLA-C*06:02 | world | 0.0878 | 0.0865 | 0.053 | 0.074 | 0.0716 | 0.074 | 0.078 | 0.0447 | 0.0932 | 0.0137 | 0.0316 | 0.0614 | 0.0105 | 0.0548 | 0.0603 | 0.1032 | 0.0697 | 0.1391 | 0.1073 | 0.0426 |
| HLA-C*07:01 | world | 0.1306 | 0.117 | 0.098 | 0.119 | 0.1161 | 0.1418 | 0.14 | 0.0101 | 0.16 | 0.024 | 0.0443 | 0.102 | 0.0073 | 0.0354 | 0.0917 | 0.1476 | 0.1172 | 0.1039 | 0.0866 | 0.0321 |
| HLA-C*07:02 | asia | 0.0683 | 0.0713 | 0.1 | 0.145 | 0.0664 | 0.0761 | 0.073 | 0.1944 | 0.1413 | 0.2894 | 0.1273 | 0.1214 | 0.1218 | 0.0822 | 0.1366 | 0.0739 | 0.151 | 0.1078 | 0.1294 | 0.1553 |
| HLA-C*07:04 | world | 0.0083 | 0.0072 | 0.006 | 0.011 | 0.0051 | 0.0091 | 0.011 | 0.0064 | 0.0154 | 0.0189 | 0.0076 | 0.0055 | 0.0094 | 0.009 | 0.0048 | 0.0111 | 0.0116 | 0.0155 | 0.0218 | 0.014 |
| HLA-C*08:01 | world | 0.0011 | 0.0011 | 0.028 | 0.014 | 0.0015 | 0.0021 | 0.003 | 0.1043 | 0.0004 | 0.1002 | 0.0931 | 0.0149 | 0.0682 | 0.0738 | 0.0362 | 0.0011 | 0.0222 | 0.0283 | 0.0499 | 0.1644 |
| HLA-C*08:02 | occident | 0.0368 | 0.034 | 0.025 | 0.035 | 0.0329 | 0.0458 | 0.037 | 0.0012 | 0.0385 | 0.0039 | 0.0119 | 0.0526 | 0.0019 | 0.0124 | 0.0512 | 0.0409 | 0.0286 | 0.0019 | 0.003 | 0.0006 |
| HLA-C*12:02 | asia | 0.0028 | 0.0011 | 0.007 | 0.012 | 0.0044 | 0.0115 | 0.022 | 0.0307 | 0.0087 | 0.0122 | 0.0163 | 0.0116 | 0.1 | 0.0268 | 0.0112 | 0.033 | 0.0048 | 0.0813 | 0.0643 | 0.0189 |
| HLA-C*12:03 | occident | 0.0183 | 0.0149 | 0.02 | 0.035 | 0.0169 | 0.0438 | 0.041 | 0.0176 | 0.0486 | 0.0051 | 0.0253 | 0.0415 | 0.0019 | 0.0056 | 0.0393 | 0.0911 | 0.0265 | 0.0491 | 0.0413 | 0.0143 |
| HLA-C*14:02 | world | 0.0155 | 0.0165 | 0.01 | 0.011 | 0.0185 | 0.0246 | 0.029 | 0.0406 | 0.0127 | 0.0135 | 0.0085 | 0.0154 | 0.0781 | 0.0823 | 0.0095 | 0.0236 | 0.0135 | 0.0336 | 0.0373 | 0.0225 |
| HLA-C*15:02 | world | 0.0044 | 0.0054 | 0.024 | 0.036 | 0.0064 | 0.0375 | 0.04 | 0.0256 | 0.0223 | 0.0681 | 0.058 | 0.0393 | 0.0196 | 0.0193 | 0.0355 | 0.0372 | 0.0435 | 0.1077 | 0.0829 | 0.0186 |
| HLA-C*16:01 | africa | 0.0914 | 0.0969 | 0.018 | 0.036 | 0.0966 | 0.085 | 0.083 | 0.0002 | 0.0338 | 0.0027 | 0.0151 | 0.0503 | 0.0016 | 0.0001 | 0.0473 | 0.0169 | 0.0281 | 0.0007 | 0.0013 | 0.0003 |
| HLA-C*17:01 | africa | 0.0715 | 0.0681 | 0.002 | 0.012 | 0.0679 | 0.0299 | 0.029 | 0.0005 | 0.0088 | 0.0014 | 0.0084 | 0.02 | 0.0007 | 0.0002 | 0.0144 | 0.0338 | 0.0082 | 0.0039 | 0.0043 | 0.0007 |

**Supplemental table 7: list of SARS-COV2 protein sequences used for the immunoinformatics prediction of MHC class I prediction:** SARS-COV2 protein identifiers found in the proteome of the virus and used to performed immunopeptidome prediction

| **number** | **Sequence identifier** | **virus** |
| --- | --- | --- |
| 1 | YP_009742617.1_nsp10 | Severe acute respiratory syndrome coronavirus 2 |
| 2 | YP_009742616.1_nsp9 | Severe acute respiratory syndrome coronavirus 2 |
| 3 | YP_009742615.1_nsp8 | Severe acute respiratory syndrome coronavirus 2 |
| 4 | YP_009742614.1_nsp7 | Severe acute respiratory syndrome coronavirus 2 |
| 5 | YP_009742613.1_nsp6 | Severe acute respiratory syndrome coronavirus 2 |
| 6 | YP_009742612.1_3C-like_proteinase | Severe acute respiratory syndrome coronavirus 2 |
| 7 | YP_009742611.1_nsp4 | Severe acute respiratory syndrome coronavirus 2 |
| 8 | YP_009742610.1_nsp3 | Severe acute respiratory syndrome coronavirus 2 |
| 9 | YP_009742609.1_nsp2 | Severe acute respiratory syndrome coronavirus 2 |
| 10 | YP_009742608.1_leader_protein | Severe acute respiratory syndrome coronavirus 2 |
| 11 | YP_009725318.1_ORF7b | Severe acute respiratory syndrome coronavirus 2 |
| 12 | YP_009725312.1_nsp11 | Severe acute respiratory syndrome coronavirus 2 |
| 13 | YP_009725311.1_2'-O-ribose_methyltransferase | Severe acute respiratory syndrome coronavirus 2 |
| 14 | YP_009725310.1_endoRNAse | Severe acute respiratory syndrome coronavirus 2 |
| 15 | YP_009725309.1_3'-to-5'_exonuclease | Severe acute respiratory syndrome coronavirus 2 |
| 16 | YP_009725308.1_helicase | Severe acute respiratory syndrome coronavirus 2 |
| 17 | YP_009725307.1_RNA-dependent_RNA_polymerase | Severe acute respiratory syndrome coronavirus 2 |
| 18 | YP_009725306.1_nsp10 | Severe acute respiratory syndrome coronavirus 2 |
| 19 | YP_009725305.1_nsp9 | Severe acute respiratory syndrome coronavirus 2 |
| 20 | YP_009725304.1_nsp8 | Severe acute respiratory syndrome coronavirus 2 |
| 21 | YP_009725303.1_nsp7 | Severe acute respiratory syndrome coronavirus 2 |
| 22 | YP_009725302.1_nsp6 | Severe acute respiratory syndrome coronavirus 2 |
| 23 | YP_009725301.1_3C-like_proteinase | Severe acute respiratory syndrome coronavirus 2 |
| 24 | YP_009725300.1_nsp4 | Severe acute respiratory syndrome coronavirus 2 |
| 25 | YP_009725299.1_nsp3 | Severe acute respiratory syndrome coronavirus 2 |
| 26 | YP_009725298.1_nsp2 | Severe acute respiratory syndrome coronavirus 2 |
| 27 | YP_009725297.1_leader_protein | Severe acute respiratory syndrome coronavirus 2 |
| 28 | YP_009725295.1_ORF1a_polyprotein | Severe acute respiratory syndrome coronavirus 2 |
| 29 | YP_009725255.1_ORF10_protein | Severe acute respiratory syndrome coronavirus 2 |
| 30 | YP_009724397.2_nucleocapsid_phosphoprotein | Severe acute respiratory syndrome coronavirus 2 |
| 31 | YP_009724396.1_ORF8_protein | Severe acute respiratory syndrome coronavirus 2 |
| 32 | YP_009724395.1_ORF7a_protein | Severe acute respiratory syndrome coronavirus 2 |
| 33 | YP_009724394.1_ORF6_protein | Severe acute respiratory syndrome coronavirus 2 |
| 34 | YP_009724393.1_membrane_glycoprotein | Severe acute respiratory syndrome coronavirus 2 |
| 35 | YP_009724392.1_envelope_protein | Severe acute respiratory syndrome coronavirus 2 |
| 36 | YP_009724391.1_ORF3a_protein | Severe acute respiratory syndrome coronavirus 2 |
| 37 | YP_009724390.1_surface_glycoprotein | Severe acute respiratory syndrome coronavirus 2 |
| 38 | YP_009724389.1_ORF1ab_polyprotein | Severe acute respiratory syndrome coronavirus 2 |

**Supplemental table 8: table with numbers of 9 mers peptides classed by binding type for each HLA allele.** Number of peptides classed according their affinity binding to MHC class pocket and summarized for each 69 more prevalent alleles

| **HLA allele** | **weak (500-5000 nM)** | **regular (50-500 nM)** | **strong (0-50 nM)** |
| --- | --- | --- | --- |
| HLA-A*01:01 | 511 | 101 | 24 |
| HLA-A*02:01 | 1428 | 553 | 375 |
| HLA-A*02:06 | 2497 | 994 | 471 |
| HLA-A*03:01 | 756 | 344 | 93 |
| HLA-A*11:01 | 899 | 450 | 244 |
| HLA-A*23:01 | 1026 | 371 | 79 |
| HLA-A*24:02 | 788 | 283 | 54 |
| HLA-A*25:01 | 394 | 58 | 3 |
| HLA-A*26:01 | 648 | 156 | 24 |
| HLA-A*29:02 | 1251 | 467 | 183 |
| HLA-A*30:01 | 3363 | 804 | 159 |
| HLA-A*30:02 | 2202 | 429 | 89 |
| HLA-A*31:01 | 1314 | 412 | 153 |
| HLA-A*32:01 | 1535 | 335 | 88 |
| HLA-A*33:03 | 860 | 359 | 115 |
| HLA-A*68:01 | 1400 | 528 | 322 |
| HLA-A*68:02 | 2243 | 654 | 302 |
| HLA-A*74:01 | 630 | 84 | 0 |
| HLA-B*07:02 | 697 | 136 | 41 |
| HLA-B*08:01 | 1281 | 230 | 35 |
| HLA-B*13:01 | 1714 | 207 | 6 |
| HLA-B*13:02 | 603 | 27 | 0 |
| HLA-B*14:02 | 648 | 41 | 3 |
| HLA-B*15:01 | 1769 | 599 | 183 |
| HLA-B*15:02 | 1541 | 543 | 109 |
| HLA-B*15:25 | 2551 | 808 | 394 |
| HLA-B*18:01 | 678 | 132 | 34 |
| HLA-B*27:02 | 87 | 5 | 0 |
| HLA-B*27:05 | 521 | 149 | 28 |
| HLA-B*35:01 | 1345 | 438 | 145 |
| HLA-B*35:03 | 434 | 58 | 5 |
| HLA-B*37:01 | 665 | 95 | 0 |
| HLA-B*38:01 | 383 | 93 | 2 |
| HLA-B*39:01 | 1103 | 234 | 58 |
| HLA-B*40:01 | 366 | 114 | 79 |
| HLA-B*40:02 | 617 | 263 | 75 |
| HLA-B*44:02 | 358 | 107 | 17 |
| HLA-B*44:03 | 335 | 104 | 20 |
| HLA-B*46:01 | 318 | 13 | 0 |
| HLA-B*48:01 | 311 | 40 | 2 |
| HLA-B*49:01 | 471 | 106 | 3 |
| HLA-B*50:01 | 538 | 155 | 19 |
| HLA-B*51:01 | 362 | 47 | 5 |
| HLA-B*52:01 | 656 | 11 | 0 |
| HLA-B*53:01 | 742 | 125 | 34 |
| HLA-B*55:01 | 290 | 45 | 7 |
| HLA-B*56:01 | 327 | 78 | 15 |
| HLA-B*57:01 | 827 | 191 | 48 |
| HLA-B*58:01 | 1566 | 343 | 118 |
| HLA-B*58:02 | 125 | 7 | 0 |
| HLA-C*01:02 | 651 | 80 | 11 |
| HLA-C*02:02 | 1730 | 345 | 18 |
| HLA-C*03:02 | 2481 | 846 | 351 |
| HLA-C*03:03 | 1546 | 540 | 209 |
| HLA-C*03:04 | 1546 | 540 | 209 |
| HLA-C*04:01 | 151 | 15 | 0 |
| HLA-C*05:01 | 625 | 126 | 53 |
| HLA-C*06:02 | 1082 | 130 | 14 |
| HLA-C*07:01 | 1347 | 122 | 6 |
| HLA-C*07:02 | 1419 | 270 | 20 |
| HLA-C*07:04 | 350 | 1 | 0 |
| HLA-C*08:01 | 612 | 88 | 5 |
| HLA-C*08:02 | 544 | 99 | 35 |
| HLA-C*12:02 | 2363 | 536 | 57 |
| HLA-C*12:03 | 2741 | 857 | 213 |
| HLA-C*14:02 | 3044 | 848 | 282 |
| HLA-C*15:02 | 1594 | 421 | 53 |
| HLA-C*16:01 | 2786 | 967 | 282 |
| HLA-C*17:01 | 1627 | 432 | 41 |
